# Sox2^+^ cells in Sonic Hedgehog-subtype medulloblastoma resist p53-mediated cell-cycle arrest response and drive therapy-induced recurrence

**DOI:** 10.1093/noajnl/vdz027

**Published:** 2019-09-23

**Authors:** Daniel M Treisman, Yinghua Li, Brianna R Pierce, Chaoyang Li, Andrew P Chervenak, Gerald J Tomasek, Guillermina Lozano, Xiaoyan Zheng, Marcel Kool, Yuan Zhu

**Affiliations:** 1 Cellular and Molecular Biology Graduate Program, Ann Arbor, Michigan; 2 Department of Internal Medicine, University of Michigan Medical School, Ann Arbor, Michigan; 3 Gilbert Family Neurofibromatosis Institute, Washington, DC; 4 Center for Cancer and Immunology Research, Washington, DC; 5 Center for Neuroscience Research, Children's National Medical Center, Washington, DC; 6 Department of Molecular Genetics, Section of Cancer Genetics, The University of Texas MD Anderson Cancer Center, Houston, Texas; 7 Department of Anatomy and Cell Biology, The GW School of Medicine and Health Sciences, The GW Cancer Center, Washington, DC; 8 Division of Pediatric Neurooncology, German Cancer Research Center (DKFZ) and German Cancer Consortium (DKTK), Heidelberg, Germany

**Keywords:** apoptosis, granule cell precursor, neural precursor, *p53*, Sonic Hedgehog medulloblastoma

## Abstract

**Background:**

High-intensity therapy effectively treats most *TP53* wild-type (*TP53*-WT) Sonic Hedgehog-subgroup medulloblastomas (SHH-MBs), but often cause long-term deleterious neurotoxicities in children. Recent clinical trials investigating reduction/de-escalation of therapy for *TP53*-WT SHH-MBs caused poor overall survival. Here, we investigated whether reduced levels of p53-pathway activation by low-intensity therapy potentially contribute to diminished therapeutic efficacy.

**Methods:**

Using mouse SHH-MB models with different p53 activities, we investigated therapeutic efficacy by activating p53-mediated cell-cycle arrest versus p53-mediated apoptosis on radiation-induced recurrence.

**Results:**

Upon radiation treatment, p53^WT^-mediated apoptosis was sufficient to eliminate all SHH-MB cells, including Sox2^+^ cells. The same treatment eliminated most Sox2^−^ bulk tumor cells in SHH-MBs harboring *p53*^R172P^, an apoptosis-defective allele with cell-cycle arrest activity, via inducing robust neuronal differentiation. Rare quiescent Sox2^+^ cells survived radiation-enhanced p53^R172P^ activation and entered a proliferative state, regenerating tumors. Transcriptomes of Sox2^+^ cells resembled quiescent Nestin-expressing progenitors in the developing cerebellum, expressing Olig2 known to suppress p53 and p21 expression. Importantly, high *SOX2* expression is associated with poor survival of all four SHH-MB subgroups, independent of *TP53* mutational status.

**Conclusions:**

Quiescent Sox2^+^ cells are efficiently eliminated by p53-mediated apoptosis, but not cell-cycle arrest and differentiation. Their survival contributes to tumor recurrence due to insufficient p53-pathway activation.

**Importance of the Study**

*TP53*-WT SHH-MBs have excellent prognosis, but long-term neurotoxicities are common. De-escalation of therapy was proposed to reduce neurotoxicities while retaining efficacy. However, recent clinical trials found that de-escalation of therapy instead caused poor overall survival. We hypothesize that therapeutic efficacy in *TP53*-WT SHH-MBs and neurotoxicities in the developing brain following standard high-intensity treatment are caused by a common mechanism—p53-mediated apoptosis. We found that radiation-enhanced p53-mediated apoptosis eliminated all SHH-MB cells and prevented tumor recurrence. Radiation-enhanced p53-mediated cell-cycle arrest, despite inducing neuronal differentiation in bulk tumor cells, failed to eliminate Sox2^+^ SHH-MB cells with transcriptomic similarity to quiescent Nestin-expressing progenitors. Importantly, high *SOX2* expression is associated with poor survival of all four SHH-MB subgroups, independent of *TP53* mutational status. Thus, our study suggests that the failure in eliminating SOX2^+^ cells may contribute to tumor recurrence, providing insights into patient stratification in future de-escalation of therapy trials.

**Key Points**

1. Quiescent Sox2^+^ SHH-MB cells resist p53-mediated cell-cycle arrest, but not apoptosis.
2. Sox2^+^ SHH-MB cells express Olig2 and are more resistant to p53-pathway activation.
3. High *SOX2* expression is associated with poor survival of *TP53*-WT SHH-MBs.

Medulloblastoma (MB) is the most common malignant brain tumor in children, 30% of which are Sonic Hedgehog-subgroup MB (SHH-MB).^1–3^ Prior human and mouse research have demonstrated that SHH-MBs arise from the granule cell precursor (GCP) lineage in the developing cerebellum.^1,4–7^ GCPs are a neuronal-restricted progenitor population transiently located in the external granular layer (EGL).^8^ During development, SHH signaling drives GCP proliferation, which then migrate into the internal granular layer (IGL) and differentiate into granule cells.^8,9^ Genetic alterations in the components of the SHH signaling pathway, including *PTCH1*, *SMOOTHENED*, *SUFU*, *GLI2*, or *N-MYC*, cause aberrant SHH-pathway activation, driving SHH-MB formation.^1,2,4,5^ Current standard treatment, including surgery, high-intensity radiation and chemotherapy, leads to 70%–80% survival for average-risk SHH-MBs, which frequently retain wild-type *TP53* alleles (*TP53*-WT SHH-MBs).^2,10,11^ However, standard high-intensity therapies often cause devastating side effects including neurocognitive, neuroendocrine, and motor deficits, diminishing the quality of life for long-term survivors.^10^

Clinical de-escalation of therapy trials, including elimination of radiation and reduced chemotherapy, has been proposed for MBs in infants and young children to avoid neurocognitive toxicities.^12,13^ However, recent de-escalation trials were suspended due to poor overall survival.^14^ These clinical observations suggest that a therapeutic threshold(s), reached by standard high-intensity therapeutic protocols, was not reached by de-escalation protocols. Given that most *TP53*-mutant SHH-MBs are in the SHHα subgroup and highly resistant to standard high-intensity therapy,^2,9–11^ the critical therapeutic threshold could be activation of p53-mediated tumor suppressive responses, including apoptosis, cell-cycle arrest, and senescence.^15^ Consistently, genetic studies using mouse models have demonstrated that disruption of p53-mediated apoptosis confers radiation resistance to SHH-MBs.^16,17^ Moreover, radiation exposure induces p53-mediated apoptosis in the developing cerebellum, leading to neurological damages.^18^ Together, we propose a model wherein activation of p53-mediated apoptosis by standard high-intensity therapy overcomes a therapeutic threshold to eliminate SHH-MB cells and prevent recurrence, but p53-mediated apoptosis may concurrently cause therapy-associated neurotoxicities.^12,13,18^ However, a recent study suggested that p53-mediated cell-cycle arrest and senescence, but not apoptosis, are sufficient to suppress SHH-MB formation.^19^ This study suggests that de-escalation of therapy may reach a lower therapeutic threshold that activates p53-mediated cell-cycle arrest and senescence, while avoiding neurotoxic p53-mediated apoptosis.

Here, we used genetically engineered mouse (GEM) models of SHH-MBs to compare therapeutic efficacy of p53-mediated cell-cycle arrest versus p53-mediated apoptosis during SHH-MB formation and radiation-induced recurrence (Figure 1A). We show that p53-mediated cell-cycle arrest response induces neuronal differentiation of SHH-MB cells, driving bulk tumor cells out of the tumor bed and dramatically reducing tumor volume. In contrast to apoptosis, however, p53-mediated cell-cycle arrest and neuronal differentiation failed to completely eliminate a previously described quiescent Sox2^+^ stem cell-like population.^20,21^ Following radiation treatment, Sox2^+^ cells entered the cell cycle and regenerated tumors. Furthermore, we provided a molecular mechanism by which Sox2^+^ SHH-MB cells are resistant to p53/p21-mediated cell-cycle arrest response via Olig2 expression. Importantly, we showed that high *SOX2* expression is associated with poor survival in all four SHH-MB subgroups, independent of *TP53* mutational status. Thus, our study provides important insights into patient stratification in the design of future de-escalation of therapy trials.

**Figure. 1:**
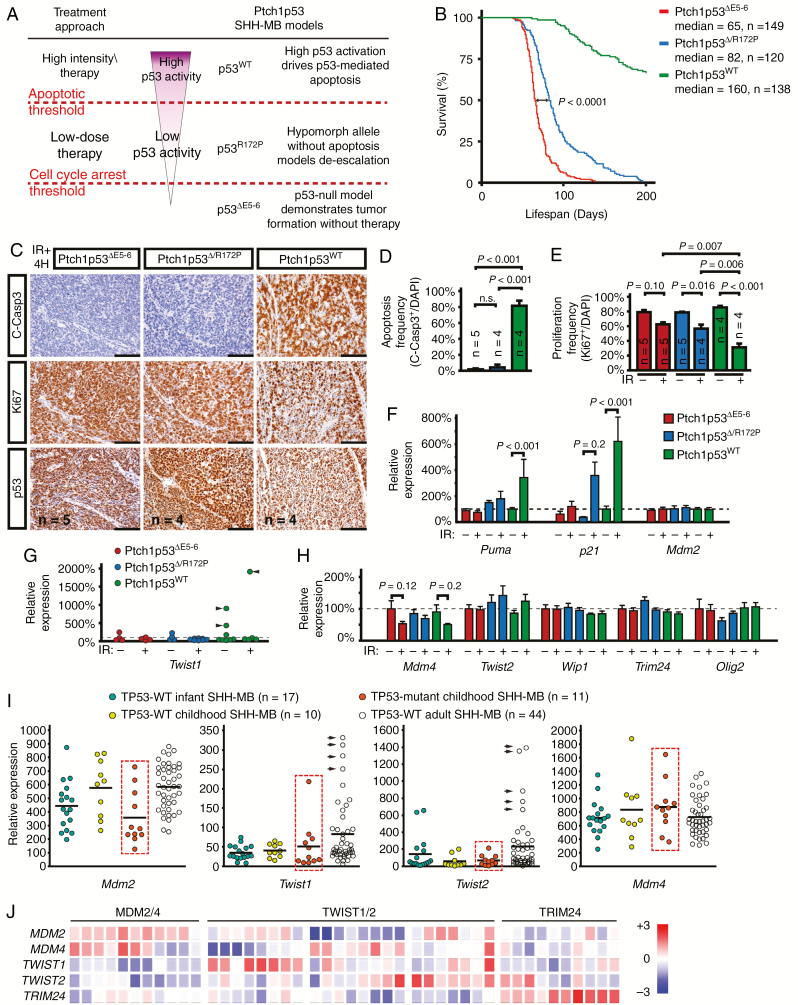
Three SHH-MB models with different p53 activities. (A) A graphic shows the relationship between existing standard high-intensity therapy versus de-escalation of therapy, p53 activation in SHH-MBs, and the p53 mutant SHH-MB models are intended to mimic these treatments. (B) Kaplan–Meier survival curves (*P*-value, Log-rank [Mantel-Cox] test) of *Ptch1*^*+/−*^*p53*^ΔE5-6/ΔE5-6^, *Ptch1*^+/−^*p53*^ΔE5-6/R172P^, and *Ptch1*^+/−^*p53*^WT/WT^ SHH-MB models are shown. (C–E) Staining of cleaved Caspase-3 (C-Casp3), Ki67 and p53 in SHH-MBs 4 h after 20 Gray radiation treatment (C). The apoptotic (D) and proliferation (E) index was quantified. (F–H) qRT-PCR was performed for p53 transcriptional targets (F) and p53-repressor elements (G and H) in un-irradiated and irradiated MBs. Fold change was normalized against un-irradiated *Ptch1*^*+/−*^*p53*^ΔE5-6^ MBs (*p53*-null) (dotted line). Arrowheads denote increased expression (G). (I) Relative expression of p53 repressor elements from human SHH-MBs sub-grouped by different ages. Red boxes label *TP53*-mutant SHH-MBs. Arrowheads denote increased expression. (J) A heatmap shows alterations of p53 repressor elements in adult human SHH-MBs. Relative expression displayed by a color scale from high (red) to low (blue). Alterations were grouped by the most altered gene(s)—*MDM2*/*4*, *TWIST1*/*2*, or *TRIM24*. Scale bars: 100 μm.

## Materials and Methods

Details are available in Supplementary Materials and Methods.

### Mouse Models and Radiation Treatment

Mice carrying a floxed *p53* (*p53*^E5-6^) allele and hGFAP-cre were described previously.^22,23^ The *p53*^R172P^ mouse strain^24^ was crossed into the hGFAP-cre;*p53*^E5-6/E5-6^ background. The *Ptch1*^*+/−*^ model^25^ was maintained on a 129 Svj/C57Bl6 mixed background prior to crossing into the hGFAP-cre; *p53*^E5-6/E5-6^ colony. Littermates were used within experimental procedures as often as possible to minimize variance. Mice with neurological symptoms were either collected or treated using a RadSource 2000. For adult mice, a Mouse Irradiation Fixture and Head Shield (Braintree Scientific Inc.) was used to limit radiation exposure to the head and neck. All mice in this study were cared for according to guidelines approved by the Animal Care and Use Committee of the University of Michigan as well as the Institutional Animal Care and Use Committee of Children's National Medical Center.

### Histology, IHC, and Western Blotting

Mice were collected as described previously.^22,23^ Tumors were classified using the WHO guidebook for MB as diagnostic criteria, based on the most severe region observed, and lesions were assessed based on previously established criteria.^19,26^ Immunohistochemistry and immunofluorescence were performed as described previously.^22^ For western blotting, samples were prepared as described previously.^23^

### Genetic Analysis and Next Generation Sequencing

Total RNA and genomic DNA was isolated from MBs and cerebellum using the AllPrep DNA/RNA Mini Kit (Qiagen), and cDNA synthesis was performed using QuantiTect Reverse Transcription Kit (Qiagen). The cDNA product, following quality assessment and measurement of concentration, was used to sequence exons of the *p53* allele, as described previously.^22^ Amplified PCR products were submitted to the University of Michigan DNA sequencing core or Genewiz L.L.C. for Sanger sequencing. Genomic DNA samples were then analyzed by next generation sequencing to detect point mutants in *Ptch1* and *p53* as described previously.^23^

### Microarray Analysis

For gene expression analyses, we used previously published microarray data sets for human SHH MBs (GSE10327; GSE37418; GSE49243; GSE85218),^1,2,27,28^ mouse Sox2^+^ and Sox2^−^ MB cells (GSE48766),^21^ and mouse Atoh1^+^ and Nestin^+^ cerebellar precursor cells plus mouse MB tumors (GSE50824).^29^ For data analysis and data visualization, we have used the R2: Genomics Analysis and Visualization Platform (http://hgserver1.amc.nl/cgi-bin/r2/main.cgi).

### Statistical Analysis

Data were analyzed and statistics performed using Graphpad Prism 6. Kaplan–Meier survival curves were compared using the Mantel-Cox test. Significance was calculated using either Student's 2-tailed *t*-test or ANOVA with Bonferroni's multiple comparisons test. Data were presented as mean ± SEM.

## Results

### Construction of Three GEM SHH-MB Models With Different p53 Activities

To investigate therapeutic efficacy of differentially activating p53-mediated apoptosis versus cell-cycle arrest thresholds, we constructed three *Ptch1* loss-driven SHH-MB models carrying a *p53* wild-type (*p53*^WT^ or *p53*^+^) or an apoptosis-defective *p53*^R172P^ allele (equivalent to *TP53*^R175P^ in humans) on a *p53 c*onditional *k*nock*o*ut (CKO) background.^22–25^ The *p53*^R172P^ allele retains partial activity of p53-mediated cell-cycle arrest responses by inducing p21 expression, but lacks apoptosis-inducing activity.^24,25^ The *p53*CKO model induces an in-frame deletion in the DNA binding domain (DBD, exons 5 and 6) of p53 (p53^∆E5-6^) driven by a neural-specific Cre transgenic line under the control of human glial fibrillary acidic protein (hGFAP) promoter (hGFAP-cre*;p53*^E5-6/E5-6^ or *p53*^∆E5-6^CKO).^22,23^ The hGFAP-cre driver is expressed in radial glial progenitors in the developing cerebellum, which give rise to most, if not all, GCPs.^4,5,22,23^ On the *p53*^∆E5-6^CKO background, a *Ptch1* heterozygous mutation (*Ptch1*^+/-^) induced MBs in 100% of *Ptch1*^+/−^*p53*^∆E5-6/ΔE5-6^ mice with median survival of 65 days (Figure 1B), providing an in vivo system to rapidly assess tumor suppressive activities of different *p53* alleles. Compared with that of *Ptch1*^+/−^*p53*^∆E5-6/ΔE5-6^ mice, survival of *Ptch1*^+/−^*p53*^ΔE5-6/R172P^ mice was extended by 26%, demonstrating p53^R172P^ tumor suppressive activities (82 days, *P* < .0001) (Figure 1B; Supplementary Figure S1A). Similar to previously published,^25^ introduction of one (or two) *p53*^WT^ allele(s) suppressed SHH-MB formation in over 60% of *Ptch1*^+/−^*p53*^∆E5-6/+^ (or *Ptch1*^+/−^*p53*^+/+^) mice (Figure 1B; Supplementary Figure S1A and B). The remaining 40% developed SHH-MBs, but significantly extended tumor latency by 146% (160 days), compared with *Ptch1*^+/−^*p53*^∆E5-6/ΔE5-6^ mice (Figure 1B; Supplementary Figure S1A). The SHH-MBs from *Ptch1*^+/−^*p53*^ΔE5-6/+^, *Ptch1*^+/−^*p53*^R172P/+^, or *Ptch1*^+/−^*p53*^+/+^ models exhibited comparable survival and penetrance, and were collectively referred to as *Ptch1*^+/−^*p53*^WT^, along with the two *p53*-mutant models, *Ptch1*^+/−^*p53*^R172P^ and *Ptch1*^+/−^*p53*^∆E5-6^ (Supplementary Figure S1B). We confirmed that SHH-MBs from all *Ptch1*^+/−^ models, regardless of *p53* status, exhibited comparable histopathological characteristics, proliferative and apoptotic index, and lineage marker expression pattern similar to human SHH-MBs (Supplementary Figure S1C–F).^23^ All these SHH-MBs exhibited aberrant activation of SHH-pathway targets *Gli1*, *Gli2*, *N-Myc*, and *Ptch1*^E7-9^ (Supplementary Figure S1G).^23^ Multiple methods, including targeted deep-sequencing of *Ptch1* alleles (300-500X coverage), revealed that nearly all (>90%) of SHH-MBs, regardless of *p53* status, exhibited loss of heterozygosity (LOH) in wild-type *Ptch1* alleles.^23^ As *Ptch1* LOH is a universal feature of all SHH-MBs, we subsequently use *Ptch1*^−/−^ to describe tumors from SHH-MB models. These results demonstrate that, compared with the *p53*^WT^ allele, p53^R172P^ exhibits less robust, but detectable tumor suppressive activities in malignant transformation of *Ptch1*^−/−^ GCPs into SHH-MBs.

### The *p53*^WT^ and *p53*^R172P^ Alleles Are Retained During *Ptch1*-Loss Driven SHH-MB Formation

Given homozygous loss of *p53* dramatically increased the penetrance and accelerated *Ptch1* loss-driven SHH-MB formation, we investigated whether *p53*^R172P^ and *p53*^WT^ alleles were, respectively, inactivated in SHH-MBs arising from *Ptch1*^+/−^*p53*^R172P^ and *Ptch1*^+/−^*p53*^WT^ models. First, we showed that both *p53*^R172P^ and *p53*^WT^ alleles were retained in SHH-MBs and SHH-MB-derived cell lines, respectively (Supplementary Figure S2A–C). Second, Sanger sequencing (*n* = 23) and targeted deep-sequencing (*n* = 20) of all SHH-MBs revealed no additional mutations in the *p53*^R172P^ or *p53*^WT^ alleles (Supplementary Figure S2D). Third and more importantly, a high-dose radiation treatment activated a potent p53 response, thereby inducing massive apoptosis and cell cycle arrest, in *Ptch1*^−/−^*p53*^WT^ SHH-MBs (Figure 1C–E). Despite robust accumulation of mutant p53 protein, no apoptosis was observed in *Ptch1*^−/−^*p53*^R172P^ or *Ptch1*^−/−^*p53*^∆E5-6^ SHH-MBs (Figure 1C–E). Of note, *Ptch1*^−/−^*p53*^R172P^ SHH-MBs exhibited reduced proliferation and activation of p21, suggesting p53-mediated cell cycle arrest (Supplementary Figure S2E–G). Consistently, radiation treatment induced the expression of p53-dependent transcriptional targets for both apoptosis, *Puma*, and cell-cycle arrest, *p21*, in *Ptch1*^−/−^*p53*^WT^ SHH-MBs, but only *p21* in *Ptch1*^−/−^*p53*^R172P^ SHH-MBs (Figure 1F).^24^ As a negative control, no induction of p53 transcriptional targets was observed in *Ptch1*^−/−^*p53*^∆E5-6^ SHH-MBs (Figure 1F). Given the intact *p53*^R172P^ or *p53*^WT^ alleles in tumors, we investigated a potential alternative mechanism(s) for p53 pathway inhibition in SHH-MBs by examining the expression of other known regulators of the p53 pathway. No alteration was observed except for elevated *Twist1* expression in a small number of *Ptch1*^*−/−*^*p53*^WT^ MBs analyzed, which was independent of radiation treatment (Figure 1G and H). Importantly, *TP53*-WT SHH-MBs from infants and children also exhibited no alteration of these p53-pathway regulators compared with *TP53*-mutant SHH-MBs (Figure 1I). Of note, many *TP53*-WT SHH-MBs in adults often exhibited mutually exclusive gene expression alterations of *MDM2, MDM4, TWIST1, TWIST2*, or *TRIM24*, suggesting a potentially attenuated p53 pathway (Figure 1J). Together, these results demonstrate that the p53 pathway is not genetically disrupted during the formation of *TP53*/*p53*-WT SHH-MBs in humans and mice, and more importantly, can be activated to exhibit therapeutic effects upon radiation treatment.

### Both p53-Mediated Cell-Cycle Arrest and Apoptosis Robustly Eliminate Proliferating Tumor Cells Following Radiation Treatment

**Figure. 2:**
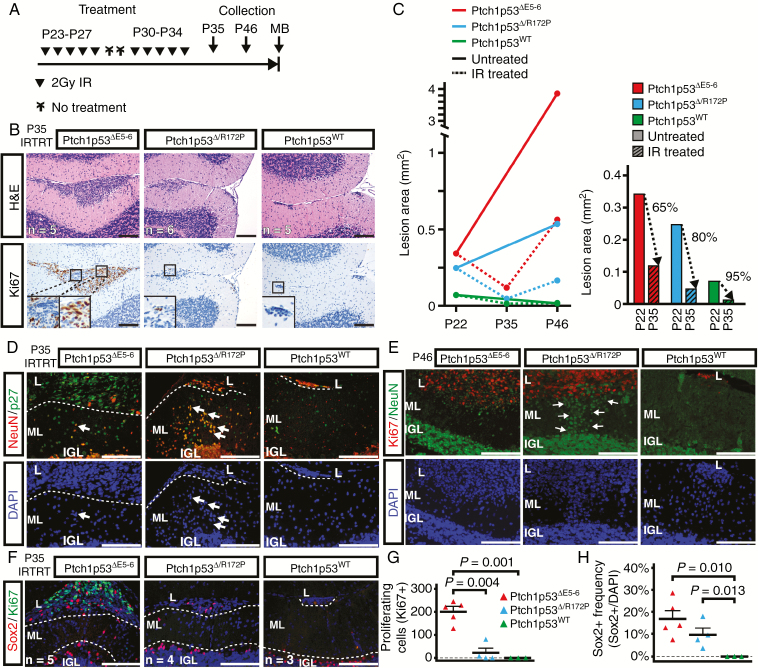
Radiation-enhanced activation of p53^R172P^ effectively eliminates proliferative Sox2^−^ bulk tumor cells, but fails to completely eliminate quiescent Sox2^+^ cells. (A) Graphical representation of clinically relevant radiation treatment. (B) Staining of H&E and Ki67 in P35 lesions following 12-day radiation treatment are shown. Insets show boxed regions. (C) The total area of untreated and radiation-treated lesions during SHH-MB formation were quantified, and relative decrease of area following treatment at P35 is shown. (D and E) Co-labeling of NeuN with p27 (D) or Ki67 (E) shows p53-mediated cell-cycle arrest and neuronal differentiation in P35 radiation-treated lesions (D) and untreated P46 lesions (E). Arrows indicate differentiated neuronal cells in the molecular layer. (F–H) Co-labeling of Sox2 and Ki67 in radiation-treated P35 lesions (F). Dotted lines demonstrate the boundary of lesions, ML and IGL. The number of Ki67^+^ proliferating cells (G) and the frequency of Sox2^+^ cells (H) in radiation-treated P35 lesions were quantified. L = lesion; ML = molecular layer; IGL = internal granular layer. Scale bars: 100 μm.

We next assessed therapeutic efficacy of activating p53^R172P^-mediated cell-cycle arrest versus p53^WT^-mediated apoptosis in SHH-MBs. We designed a clinically relevant treatment protocol in the SHH-MB models, administering 10 fractions of 2 Gray each over 12 days, from P22 to P34, and then investigated therapeutic effects at three time points: 1 day (P35) or 12 days (P46) after the completion of radiation treatment, or long-term follow-up until tumors emerged (Figure 2A). Compared with untreated lesions at P22 (Supplementary Figure S3A–C), radiation treatment shrank lesions in all models at P35, exhibiting 65%, 80%, and 95% of reduction in SHH-MBs with *p53*^∆E5-6^, *p53*^R172P^, and *p53*^WT^ alleles, respectively (Figure 2B and C; Supplementary Figure S3D). These results suggest that radiation induces both p53-independent and p53-dependent tumor inhibitory effects. However, the radiation-treated lesions were distinctly different in these three models. Radiation-treated lesions in the *Ptch1*^+/−^*p53*^∆E5-6^ model exhibited widespread proliferation, high levels of mutant p53 expression, and maintained rare quiescent Sox2^+^ cells. Small focal regions within the proliferating *Ptch1*^+/−^*p53*^∆E5-6^ tumors morphologically resembled differentiated neurons of the IGL with the expression of the markers for differentiating cells, p27, and neurons, NeuN, but no Ki67 or p53^∆E5-6^ expression (Figure 2B and D; Supplementary Figure S3E–G). This observation suggests that radiation induces neuronal differentiation of a subset of tumor cells in a p53-independent manner. In contrast, surviving lesions in radiation-treated *Ptch1*^+/−^*p53*^R172P^ cerebella exhibited almost no proliferating cells, but predominantly comprised of morphologically IGL-like differentiated neurons with expression of p27 and NeuN (Figure 2B and D; Supplementary Figure S3E–G). More importantly, radiation-enhanced p53^R172P^ activation induced massive neuronal differentiation characterized by a large number of NeuN^+^ cells migrating out of the surviving lesions into the IGL, a feature also observed during untreated formation of *Ptch1*^−/−^*p53*^R172P^ SHH-MB (arrows, Figure 2D and E). The chain-like NeuN^+^ cells are reminiscent of differentiating GCPs migrating from the EGL to IGL during cerebellar development.^8^ Despite effectively driving tumor cells out of the cell cycle and inducing neuronal differentiation, radiation treatment failed to completely eliminate quiescent Sox2^+^ cells in *Ptch1*^−/−^*p53*^R172P^ tumors (Figure 2F–H). Strikingly, this radiation treatment protocol almost completely eliminated tumor or tumor-like cells in the *Ptch1*^+/−^*p53*^WT^ cerebella (Figure 2F–H), though apoptotic cells were no longer detected (data not shown). The rare lesion-like cells in radiation-treated *Ptch1*^+/−^*p53*^WT^ cerebella morphologically resembled differentiated neurons in the IGL with expression of p27 and NeuN, but not Sox2 (Figure 2F–H; Supplementary Figure S3F and G). These results demonstrate that radiation-induced p53^R172P^-mediated cell-cycle arrest induces massive differentiation of bulk tumor cells, but fails to completely eliminate quiescent Sox2^+^ cells. In contrast, radiation-induced p53^WT^-mediated apoptosis and cell-cycle arrest efficiently eliminate all tumor cells, including Sox2^+^ cells.

### Sox2^+^ cells regenerate tumors in the *Ptch1*^+/-^*p53*^R172P^ model following radiation treatment

**Figure. 3:**
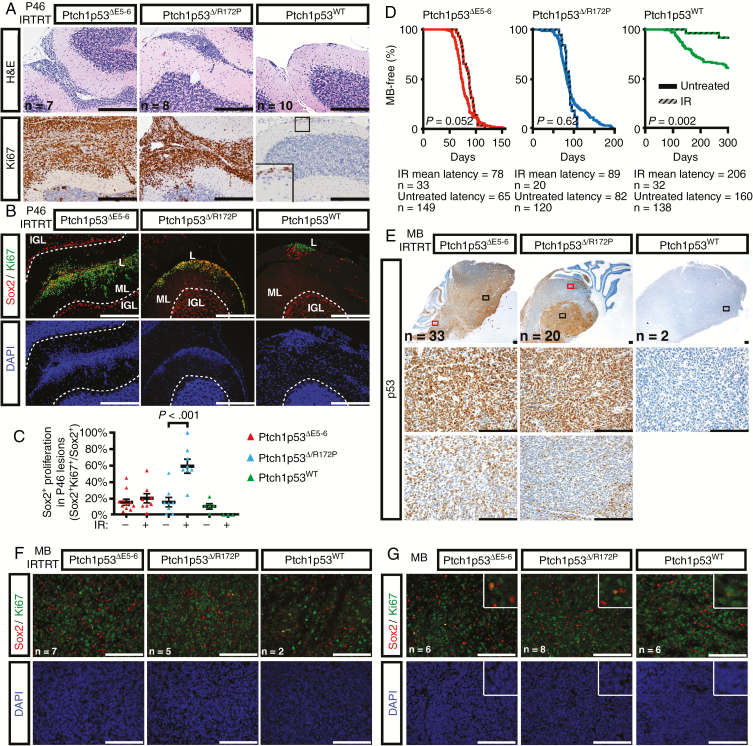
Activation of *p53*^WT^-mediated apoptosis effectively eliminates SHH-MBs, but surviving Sox2^+^ cells drive *p53*^R172P^ SHH-MB recurrence. (A) Representative images of H&E and Ki67 in P46 radiation-treated lesions are shown. Insets show high magnification of the boxed regions. (B and C) Co-labeling of Sox2 and Ki67 in radiation-treated P46 lesions (B). Dotted lines demonstrate the boundary of lesions, ML and IGL. The proliferation index of Sox2^+^ cells in radiation-treated P46 lesions (C) was quantified. (D) Kaplan–Meier survival curves (*P*-value, Log-rank [Mantel-Cox] test) of treated and untreated *Ptch1*^*+/−*^*p53*^ΔE5-6^, *Ptch1*^+/−^*p53*^R172P^, *Ptch1*^+/−^*p53*^WT^, and *Ptch1*^+/−^*p53*^WT/R172P^ models are shown. (E) Staining of p53 in SHH-MBs arising following radiation treatment are shown. Boxed regions are shown at higher magnification below. (F and G) Co-labeling of Sox2 and Ki67 in SHH-MBs arising following radiation treatment (F) and untreated SHH-MBs (G) are shown. Scale bars: 50 μm (A and B). 100 μm (E–G).

Consistent with distinct therapeutic outcomes observed at P35, surviving MB-like lesions all developed into SHH-MBs in radiation-treated *Ptch1*^+/−^*p53*^∆E5-6^ cerebella, whereas almost no evidence of tumors or proliferating cells was observed in radiation-treated *Ptch1*^+/−^*p53*^WT^ cerebella at P46 (Figure 3A; Supplementary Figure S4A–D). Unexpectedly, radiation-treated lesions in *Ptch1*^+/−^*p53*^R172P^ cerebella enlarged significantly and showed greater proliferation frequency than untreated counterparts by P46 (Figure 3A; Supplementary Figure S4A–D). Tumor regeneration was traced to Sox2^+^ cells, which, while largely quiescent in P35 radiation-treated *Ptch1*^+/−^*p53*^R172P^ cerebella (Figure 2F–H), entered the cell cycle by P46 (Figure 3B and C). The percentage of proliferating Sox2^+^Ki67^+^ cells within Sox2^+^ population of radiation-treated *Ptch1*^+/−^*p53*^R172P^ lesions reached approximately 60%, compared with 15%–20% of proliferation rate in either untreated or treated *Ptch1*^+/−^*p53*^∆E5-6^ lesions (Figure 3B and C; Supplementary Figure S4C and D). In contrast, Sox2^+^ cells were not detected in most lesions of radiation-treated *Ptch1*^+/−^*p53*^WT^ cerebella, and in rare cases, the remaining Sox2^+^ cells were not proliferating and the surrounding cells characterized by the IGL-like neuronal morphology (Figure 3B and C). Together, these results demonstrate that radiation-enhanced p53^WT^ activation efficiently eliminates all tumor cells. In contrast, radiation-enhanced p53^R172P^ activation, despite driving widespread neuronal differentiation and migration of bulk tumor cells, fails to efficiently eliminate quiescent Sox2^+^ cells, which become highly proliferative and rapidly regenerate tumors.

Consistently, radiation-treated *Ptch1*^+/−^*p53*^∆E5-6/∆E5-6^ or *Ptch1*^+/−^*p53*^∆E5-6/R172P^ mice developed SHH-MBs with complete penetrance, median survival, p53 expression, and proliferation frequency comparable to untreated SHH-MBs (Figure 3D and E; Supplementary Figure S4E and F). Of note, although we observed that Sox2^+^ cells in *Ptch1*^−/−^*p53*^R172P^ lesions survived radiation and entered the cell cycle, the Sox2^+^ cells became a rare quiescent cell population in all recurrent SHH-MBs at end stages, comparable to untreated SHH-MBs (Figure 3F and G).^20,21^ In contrast, radiation treatment dramatically reduced tumor penetrance compared with untreated *Ptch1*^+/−^*p53*^WT^ mice (38% to 9%), and tumor latency further increased by 29% in the only two treated mice (that still developed tumors) (Figure 3D). Next-generation sequencing failed to detect any *p53* mutations in these two radiation-treated SHH-MBs, suggesting some *p53*^WT^ SHH-MB cells survived radiation treatment, supported by the lack of stabilized p53 in the resultant SHH-MBs (Figure 3E). These results support the model wherein activation of p53^WT^-mediated apoptosis is the therapeutic threshold to overcome in order to eliminate Sox2^+^ SHH-MBs. In contrast, radiation-enhanced p53^R172P^ activation has no or little benefit on preventing the recurrence of SHH-MBs as a result of failing to eliminate quiescent Sox2^+^ tumor cells.

### The Transcriptome of Sox2^+^ SHH-MB Cells Resembles Developing Quiescent Nestin-Expressing Progenitors With Olig2 Expression

**Figure. 4:**
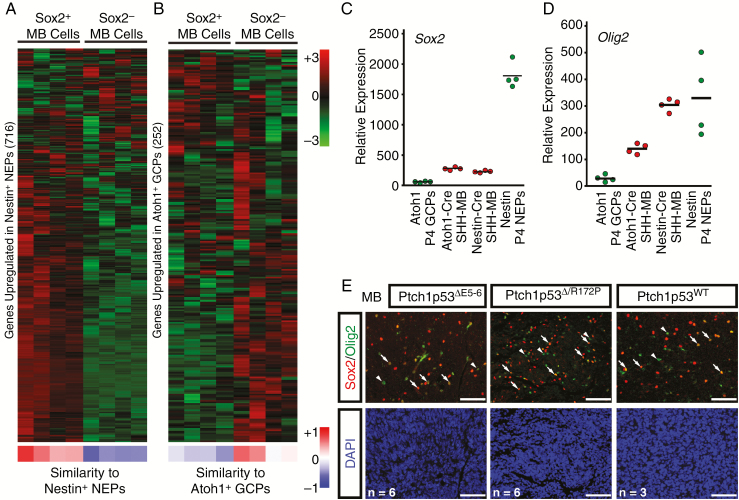
Similar transcriptional profiles of Sox2^+^ SHH-MB cells and quiescent Nestin-expressing progenitors (NEPs) identifies upregulation of Olig2, a known p53 inhibitor. (A and B) Genes identified from supervised clustering of Nestin^+^ NEPs and Atoh1^+^ GCPs were examined in SHH-MB cells. Heat map shows similar expression of 716 genes between NEPs and Sox2^+^ SHH-MB cells (A) and 504 genes between Sox2^−^ SHH-MB cells and GCPs (B). Relative expression levels and transcriptional profile comparison are displayed by a color scale from high (red) to low (green or blue, respectively). (C and D) *Sox2* (C) and *Olig2* (D) expression were compared within P4 Atoh1^+^ GCPs, SHH-MBs from Atoh1-cre*;Ptch1*^flox/flox^, SHH-MBs from Nestin-cre*;Ptch1*^flox/flox^, and P4 Nestin^+^ NEPs. (E) Co-labeling of Sox2 and Olig2 in SHH-MBs are shown. Arrows show Olig2^+^Sox2^+^ and arrowheads indicate Olig2^+^Sox2^−^ cells. Scale bars: 100 μm.

Our results suggest that Sox2^+^ SHH-MB cells resist radiation-enhanced p53^R172P^-dependent cell-cycle arrest and neuronal differentiation, which is mediated by p21 expression.^24^ To investigate the underlying mechanism, we analyzed published microarray data sets of Sox2^+^ and Sox2^−^ cells isolated from SHH-MBs in a similar *p53*-WT *Ptch1*^+/−^ model.^21^ Between these two populations, 107 genes were significantly upregulated in Sox2^+^ SHH-MB cells (Supplementary Figure S5A), whereas Sox2^−^ SHH-MB cells had 7 upregulated genes (*P* < .01) (Supplementary Figure S5B). Gene Ontology (GO) analysis found Sox2^+^ SHH-MB cells upregulated several genes involved in negative regulation of neuronal differentiation, confirming that Sox2^+^ SHH-MB cells are less differentiated with stem-cell–like characteristics (Supplementary Figure S5C). Given the quiescence and undifferentiated nature of Sox2^+^ SHH-MB cells, we next investigated whether they are similar to a recently discovered quiescent Nestin-expressing precursor (NEP) population in the GCP lineage.^29^ We therefore analyzed published data sets showing expression patterns of Nestin^+^Atoh1^−^ NEPs and Nestin^−^Atoh1^+^ GCPs.^29^ In P4 cerebella, 716 genes were upregulated in NEPs, whereas 252 were upregulated in GCPs (*P* < .001). Importantly, the majority of the 716 genes upregulated in NEPs were also upregulated in the Sox2^+^ SHH-MB cells, whereas the genes upregulated in the Atoh1^+^ GCPs were upregulated in the Sox2^−^ SHH-MB cells (Figure 4A and B; Supplementary Figure S5D and E). Likewise, most of the 107 significantly upregulated genes in the Sox2^+^ SHH-MB cells were also upregulated in NEPs, but not in GCPs or SHH-MB cells (Supplementary Figure S5A). In contrast, the genes downregulated in Sox2^+^ SHH-MB cells were also downregulated in NEPs (Supplementary Figure S5B). Comparable genome-wide expression profiles between Sox2^+^ SHH-MB cells and NEPs as well as between Sox2^−^ SHH-MB cells and GCPs suggest that a similar process occurs between tumor regeneration from Sox2^+^ to Sox2^−^ cells in radiation-treated SHH-MBs and the regeneration of GCPs from NEPs in radiation-treated developing cerebellum.^21,29,30^

One of the genes upregulated in both Sox2^+^ SHH-MB cells and Sox2^+^ NEPs, encodes transcription repressor Olig2 (Figure 4C and D). OLIG2 is a pan-glioma marker also expressed in a rare cell population within human MBs.^4^ Previous studies demonstrated that Olig2 represses p53-mediated transcriptional activities, by inhibiting acetylation of p53, and directly represses transcription of p21^31,32^. Similar to human MBs,^4^ we showed that a minor cell population in tumor lesions—including many Sox2^+^ cells—expressed Olig2 protein (Figure 4E; Supplementary Figure S5F). Specific expression of Olig2 in Sox2^+^ SHH-MB cells may provide a mechanism for the resistance to p53-dependent p21-mediated cell-cycle arrest during SHH-MB formation and radiation treatment.

### Sox2^+^ SHH-MB Cells Are Most Resistant to p53-Pathway Activation

Inhibition of p53 acetylation by Olig2 raises the possibility that Sox2^+^ SHH-MB cells are more resistant to p53-pathway activation upon radiation treatment. To test this idea, we sought to determine the mechanism for selective accumulation of mutant p53^ΔE5-6^ protein in tumor and stressed cells. First, we showed that p53^ΔE5-6^ detection by a p53 antibody was specifically eliminated by *p53*-specific siRNAs, but not mismatched *p53* siRNAs with substitutions of three nucleotides (Supplementary Figure S6A and B). These results demonstrate that, despite deletion of the DBD, the other domains of the p53^ΔE5-6^ protein are expressed and recognized by this p53 antibody. Second, we showed that MDM2/Mdm2 could bind and degrade mutant p53 protein encoded by *TP53*^ΔE5-6^/*p53*^ΔE5-6^ in both human and mouse cells, demonstrating a mechanism for preventing p53^ΔE5-6^ accumulation in normal cells (Supplementary Figure S6C–F).^22^ Due to a lack of transcriptional activity, however, the p53^ΔE5-6^ protein disrupts the p53-Mdm2 negative feedback loop, increasing the half-life of the p53^ΔE5-6^ protein in stressed and tumor cells.^33^ Thus, these results establish selective accumulation of mutant p53^ΔE5-6^ protein as an in vivo marker for p53-pathway activation in tumor and stressed cells.

**Figure. 5:**
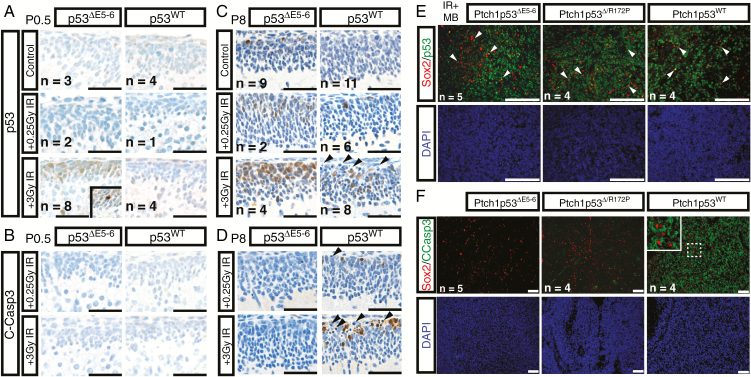
Expression of p53^∆E5-6^ protein is a marker of radiation-induced p53 activation in Sox2^−^ but not Sox2^+^ SHH-MB cells. (A–D) Representative staining images of p53 (A and C) and cleaved Caspase-3 (C-Casp3) (B and D) from P0.5 cerebella (A and B) and P8 cerebella (C and D) collected 4 h after either sham or radiation treatment are shown. Inset shows a p53-positive cell (A). Arrowheads demonstrate apoptotic nuclei (C and D). (E and F) Co-labeling of Sox2 with p53 (E) and apoptosis maker Cleaved Caspase-3 (F) in SHH-MBs are shown. Arrowheads indicate rare Sox2^+^ cells with p53 expression (E). Insets show high magnification of the boxed regions.

We next explored differential activation of p53-mediated apoptosis in GCPs at P0.5 and P8 following radiation treatment.^34,35^ As previously described, no accumulation of p53^WT^ or p53^ΔE5-6^ protein was detected in the GCPs of the cerebella of P0.5 *p53*^WT/WT^ or *p53*^∆E5-6/∆E5-6^ mice 3 h after low (0.25 Gy) or high (3 Gy) dosage radiation treatment (Figure 5A and B). After treating P8 *p53*^WT/WT^ mice with high-dosage radiation, a robust apoptotic response was accompanied by accumulation of p53^WT^ protein in some cells within the highly proliferative outer layer of the EGL (oEGL), exclusively comprised of proliferating GCPs (Figure 5C and D).^8,34,35^ Importantly, selective accumulation of p53^ΔE5-6^ protein was uniformly observed in proliferating GCPs of in the oEGL of radiation-treated *p53*^∆E5-6/∆E5-6^ mice, despite no evidence of apoptosis (Figure 5C and D). Furthermore, accumulation of p53^ΔE5-6^, not p53^WT^ protein, was detected in proliferating GCPs even after low-dosage radiation, which was insufficient to detect apoptosis in proliferating GCPs at P8 (Figure 5C and D). Thus, selective accumulation of p53^ΔE5-6^ protein provides a sensitive marker to identify proliferating GCPs that trigger radiation-induced p53-mediated apoptosis, which otherwise would be eliminated and undetectable in the presence of p53^WT^ function in vivo.^34,35^ Using the p53^ΔE5-6^ as a marker, we showed that whereas bulk tumor cells exhibited robust expression of mutant p53^∆E5-6^ protein, Sox2^+^ cells rarely expressed a detectable level of p53^∆E5-6^ protein in SHH-MBs even following high-dose radiation (Figure 5E). Furthermore, Sox2^+^ cells were relatively more resistant to p53-mediated apoptosis, despite a widespread apoptotic response in the entire MB areas of *Ptch1*^+/−^*p53*^WT^ mice under high doses of radiation treatment (Figure 5F). These observations demonstrate that, similar to P0.5 GCPs, a lack of mutant p53^∆E5-6^ accumulation in Sox2^+^ cells within *p53*-mutant SHH-MBs accurately predicts the resistance to p53-pathway activation observed in Sox2^+^ MB-stem cells within *p53*-WT SHH-MBs.

### High *SOX2* Expression Is Associated With Poor Survival of *TP53*-WT SHH-MBs

**Figure. 6:**
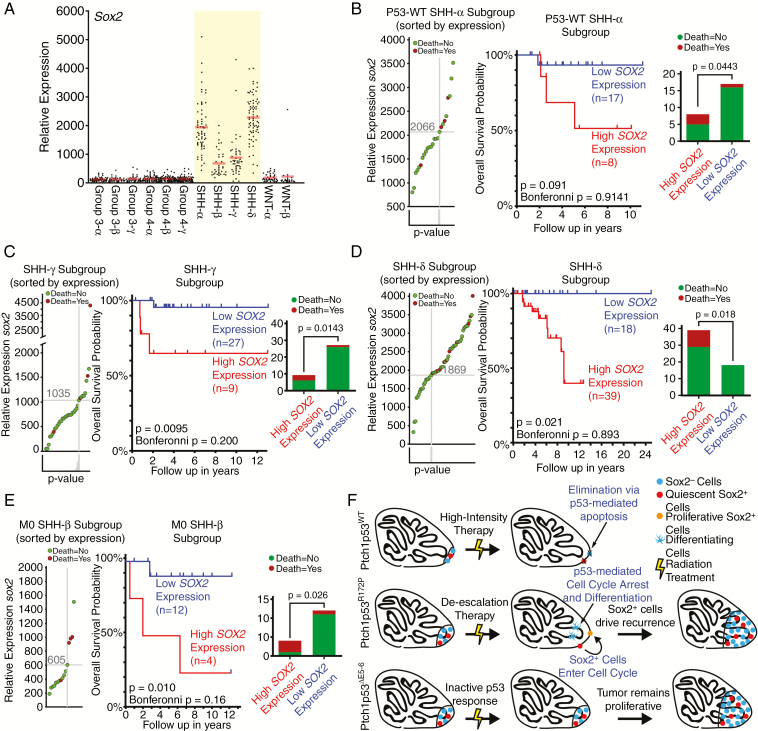
Association of *SOX2*-expression with prognosis in patients from each SHH-MB subgroup. (A) *SOX2* gene expression was analyzed for each subgroup of human MBs. (B–E) Scatterplot distribution of *SOX2* gene expression (left), survival analysis (middle), and tumor mortality (right) of patients among: *TP53*-WT SHH-MBα (B); all SHH-MBγ (C); all SHH-MBδ (D); and nonmetastatic (M0) SHH-MBβ (E) subgroup. Patients deaths from tumor recurrence/progression are represented by red dots. Live patients are represented by green dots. Multiple analyses were conducted in the *SOX2* expression datasets to find the expression threshold with the greatest significance (the lowest p-value) between *SOX2*-high and -low expression values, and the most significant is shown (*P*-value, Log-rank [Mantel-Cox] test and Bonferroni-corrected Log-rank [Mantel-Cox] test). Frequency of tumor mortality at these thresholds was analyzed (chi-square test). (F) A graphic depicting the overall role of SOX2^+^/Sox2^+^ cells in SHH-MBs.

We sought to determine whether *SOX2* expression affected patient survival in four recently identified SHH-MB subgroups.^2^ High levels of *SOX2* expression were only observed in SHH-MBs, but not WNT, Group 3 or 4 subtypes (Figure 6A). However, significant variations in *SOX2* expression were observed between the 4 SHH-MB subgroups: SHH-MBα and SHH-MBδ subgroups expressed *SOX2* at higher levels than SHH-MBβ and SHH-MBγ subgroups (Figure 6A). Accordingly, we investigated the association between *SOX2* expression and patient survival within each SHH-MB subgroup. In the SHH-MBα subgroup, *TP53* mutations are associated with poor survival; thus, we investigated the association between differential *SOX2* expression levels and survival in *TP53*-WT SHH-MBα, as well as overall (Figure 6B; Supplementary Figure S7A). Importantly, more *TP53*-WT SHH-MBα patients with high *SOX2* expression (3 of 8 [37.5%]) died than those with low levels of *SOX2* expression (1 of 17 [6%]) (Figure 6B). As *TP53* mutation is not a prognostic factor for the three non-SHH-MBα subgroups, we investigated the relation between *SOX2* expression and prognosis in each of these subgroups without excluding any tumors (Figure 6C and D). Patient death was rare in the SHH-MBδ subgroup (0/18, 0%) and the SHH-MBγ subgroup (1/27, 4%) with low levels of *SOX2* expression, whereas significantly more deaths occurred in tumors with high levels of *SOX2* expression in SHH-MBδ (10/39, 26%) and in SHH-MBγ (3 of 9, 33%) subgroup (Figure 6C and D). As the SHH-MBβ subgroup has been shown to see more frequent metastases which directly correspond to worse prognosis, we investigated *SOX2* expression levels and survival in non-metastatic (M0) SHH-MBβ, as well as overall (Figure 6E; Supplementary Figure S7B). We observed more deaths associated with high levels of *SOX2* expression (3 of 4 [75%]) than those with low levels of *SOX2* expression (1 of 12, 8.3%) in the non-metastatic SHH-MBβ subgroup (Figure 6E). These patient data demonstrate that the high *SOX2* expression corresponds to poor survival, supporting our observation that Sox2^+^ SHH-MB cells may contribute to tumor recurrence.

## Discussion

The effective treatment response frequently observed in *TP53*-WT SHH-MBs led to the widely held belief that a lower dosage therapeutic approach, which could carry less neurotoxic burden, might be clinically efficacious.^12,13^ However, recent clinical trials of de-escalation of therapy have not met with success.^14^ Our study revealed that a rare population of SOX2^+^/Sox2^+^ cells may be responsible for treatment resistance in *TP53*^WT^/*p53*^WT^ SHH-MBs. Although previous research has shown that Sox2^+^ cells are more resistant to chemotherapy compared with Sox2^−^ bulk tumor cells in SHH-MBs with wild-type *p53*^21^, the mechanism by which Sox2^+^ cells evade therapy remains unclear. We therefore investigated the mechanism by which these cells remain even when surrounding cells were eliminated. Using SHH-MB models carrying two different *p53* mutant alleles,^22–24^ we show a molecular mechanism for the resistance of Sox2^+^ cells to chemo/radiation therapy. First, we investigated relative sensitivity of Sox2^+^ versus Sox2^−^ cells to p53-pathway activation in vivo by exploring a conditional in-frame *p53* deletion mutant allele. Mutant p53^∆E5-6^ protein lacks transcriptional activity, but retains the ability to be bound and degraded by Mdm2. Thus, expression of mutant p53^∆E5-6^ protein was undetectable in most normal cells (likely due to basal Mdm2 activity). However, upon p53-pathway activation, mutant p53^∆E5-6^ protein rapidly accumulated and sustained high levels of expression in tumor cells as—only possible because of a lack of transcriptional activation of *Mdm2* and disruption of the negative feedback loop of the p53-Mdm2 regulatory axis. Consequently, we show that Sox2^+^ cells are more resistant to p53-pathway activation following radiation treatment. In addition, we propose a potential mechanism for this resistance via Olig2 expression in Sox2^+^ SHH-MB cells, which has shown to acetylate and suppress p53 as well as inhibit p21 expression.^31,32^ Second, using the apoptosis-defective *p53*^R172P^ mutant allele, we show that Sox2^+^ cells were resistant to p53-dependent p21-mediated cell-cycle arrest response, whereas radiation-enhanced p53^R172P^ activation induced massive neuronal differentiation of Sox2^−^ bulk tumor cells and drove them out of the tumor bed. The similarity in transcriptomes between Sox2^+^ SHH-MB cells and the recently identified quiescent NEPs, including Olig2 expression,^29^ provides a mechanism for the resistance of Sox2^+^ cells to stress- and therapy-induced p53-pathway activation as well as p21-mediated cell-cycle arrest response.

Despite the resistance to p53^R172P^-mediated cell-cycle arrest and neuronal differentiation, radiation-enhanced p53^WT^-mediated apoptosis efficiently eliminated both Sox2^+^ and Sox2^−^ SHH-MB cells, almost completely preventing tumor recurrence. It has been shown that p53 binding targets include both high-affinity binding sites, including cell-cycle arrest targets, and low-affinity sites, including apoptotic targets, are activated at low and high thresholds of p53-pathway activation, respectively.^15,18^ Since therapeutic activation of p53 leads to the successful treatment of SHH-MBs, we propose that the recent de-escalation trials failed to generate sufficiently high levels of p53-pathway activation to induce apoptosis, and consequently, fail to eliminate Sox2^+^ cells (Figure 6F). Following this treatment response, the Sox2^+^ cells were capable of rapidly reentering the cell cycle and generating a rapidly proliferating recurrent tumor, a behavior comparable to NEPs in the developing cerebellum, which can reenter the cell cycle following injury.^30^ These findings emphasize the need to use therapeutic approaches that can effectively eliminate quiescent Sox2^+^ cells to prevent recurrence. To corroborate this model and the importance of SOX2^+^ cells would require a clinically challenging approach involving analysis of tumor samples at multiple time points following de-escalating therapy. We sought to obtain supporting evidence by investigating the association between high *SOX2* expression and overall survival in recently published data sets from human SHH-MBs.^2^ We found a significant variation of *SOX2* expression among the four SHH-MB subgroups. Strikingly, we found that high *SOX2* expression corresponds to poor survival from all four SHH-MB subgroups. Although a larger series of patient data are required to validate these results, these observations provide the evidence supporting the model wherein SOX2^+^/Sox2^+^ SHH-MB cells are more resistant to therapy-induced activation of p53-mediated tumor suppressive responses (e.g., cell-cycle arrest and neuronal differentiation) and responsible for tumor recurrence following de-escalating therapies. Together, our study provides important insights into the stratification of children with *TP53*-WT SHH-MBs based on the levels of *SOX2* expression that may be considered for the design of future de-escalation of therapy trials.

## Supplementary Material

Supplementary material is available online at *Neuro-Oncology* (http://neuro-oncology.oxfordjournals.org/).

vdz027_suppl_Supplementary_Figure_S1

vdz027_suppl_Supplementary_Figure_S2

vdz027_suppl_Supplementary_Figure_S3

vdz027_suppl_Supplementary_Figure_S4

vdz027_suppl_Supplementary_Figure_S5

vdz027_suppl_Supplementary_Figure_S6

vdz027_suppl_Supplementary_Figure_S7

vdz027_suppl_Supplementary_Materials

## Author Contributions

D.M.T., A.P.C., and Y.Z. conceived and designed the study. D.M.T., Y.L., C.L., A.P.C., B.P., and G.J.T. performed the experiments. D.M.T. and Y.Z. analyzed the results. M.K. conducted the bioinformatics and genome-wide analysis of MBs. G.L. and X.Z. assisted with supervision and resources. Y.Z. and D.M.T. acquired the funding and wrote the manuscript with contributions from all the authors.

## Funding

This work was supported by grants from the National Institutes of Health (2P01 CA085878-10A1 and 1R01 NS053900) and Cancer Biology Training is an NIH - NCI T32 grant (5T32CA009676-22) and the Cellular and Molecular Biology is an NIH - NIGMS T32 grant (5T32GM007315-43), University of Michigan.
