# Supplementary figures and images for: Sox2+ cells in Sonic Hedgehog-subtype medulloblastoma resist p53-mediated cell-cycle arrest response and drive therapy-induced recurrence
Source: Neurooncol Adv. 2019 Sep 23;1(1):vdz027. doi: 10.1093/noajnl/vdz027 (PMC6860004; doi:10.1093/noajnl/vdz027)

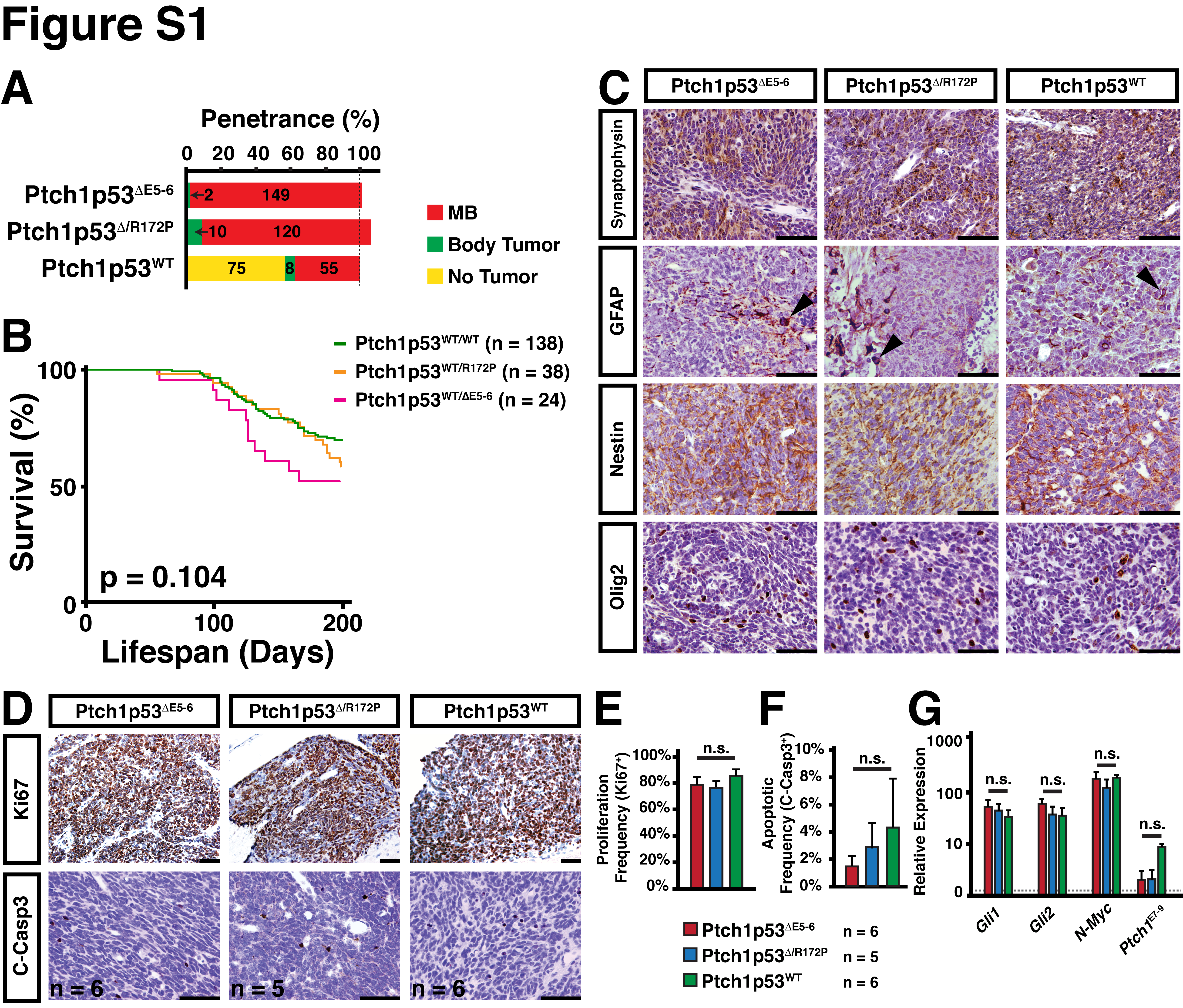

Supplement: vdz027_suppl_Supplementary_Figure_S1 [file vdz027_suppl_supplementary_figure_s1.png]

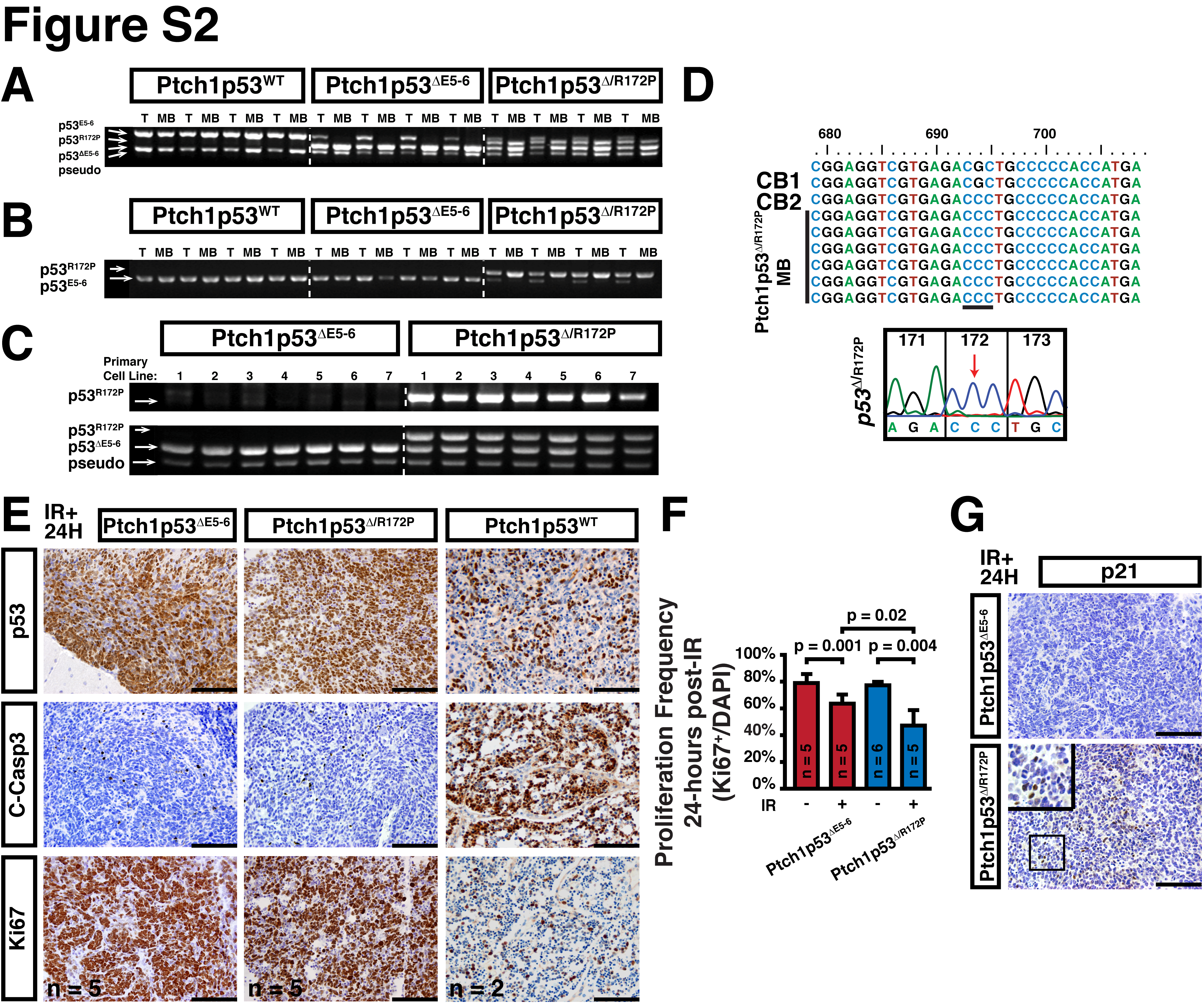

Supplement: vdz027_suppl_Supplementary_Figure_S2 [file vdz027_suppl_supplementary_figure_s2.png]

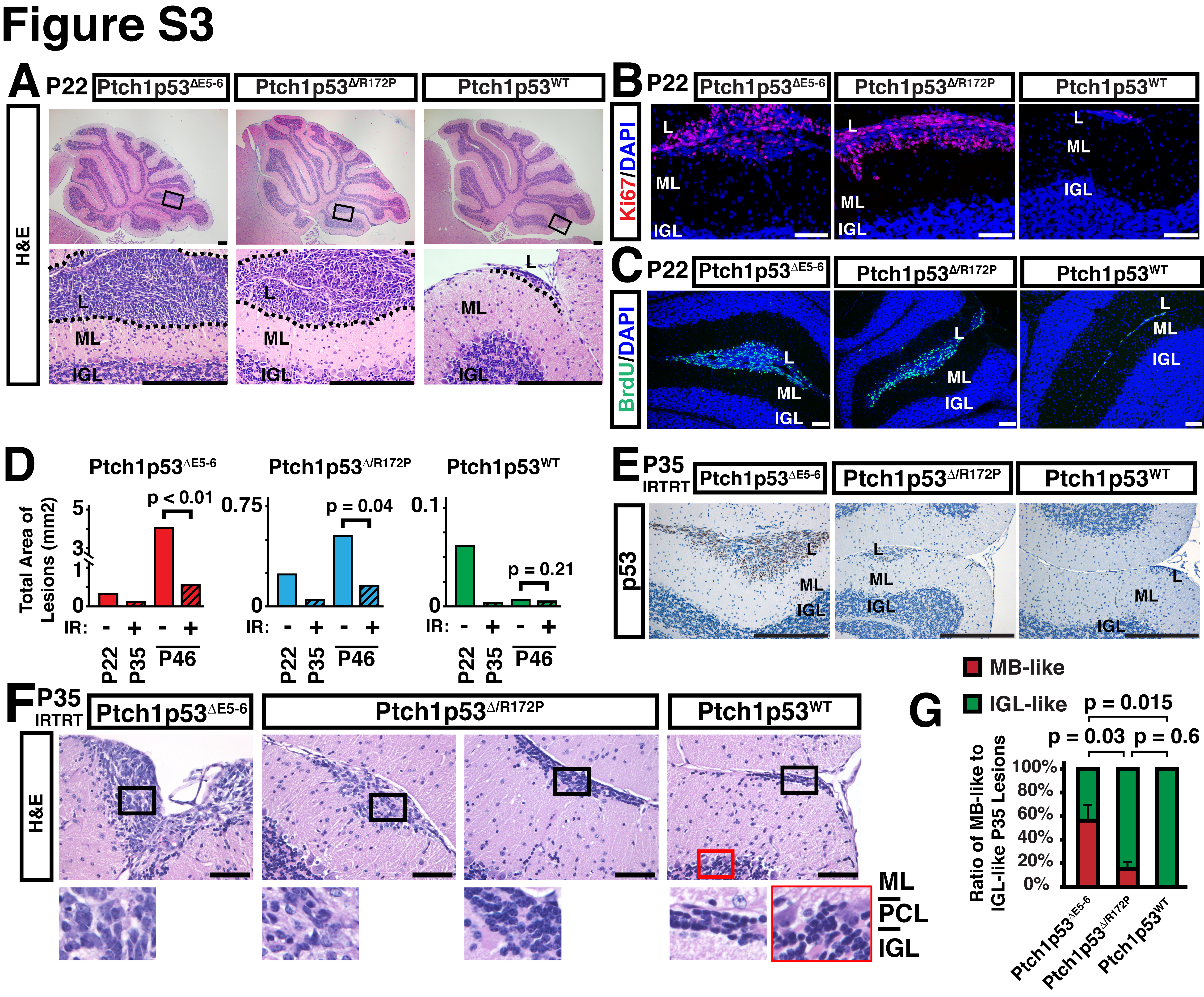

Supplement: vdz027_suppl_Supplementary_Figure_S3 [file vdz027_suppl_supplementary_figure_s3.png]

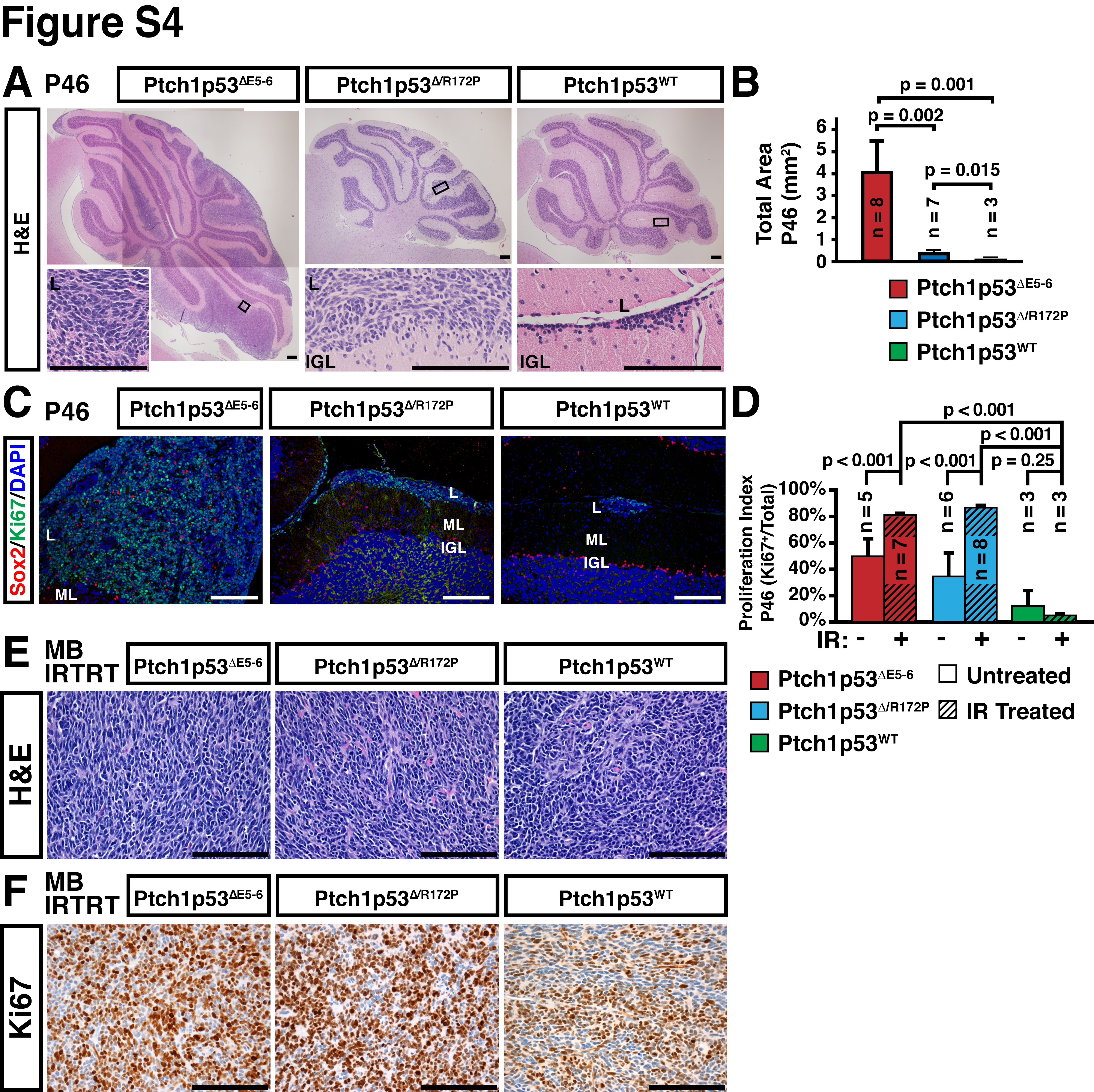

Supplement: vdz027_suppl_Supplementary_Figure_S4 [file vdz027_suppl_supplementary_figure_s4.png]

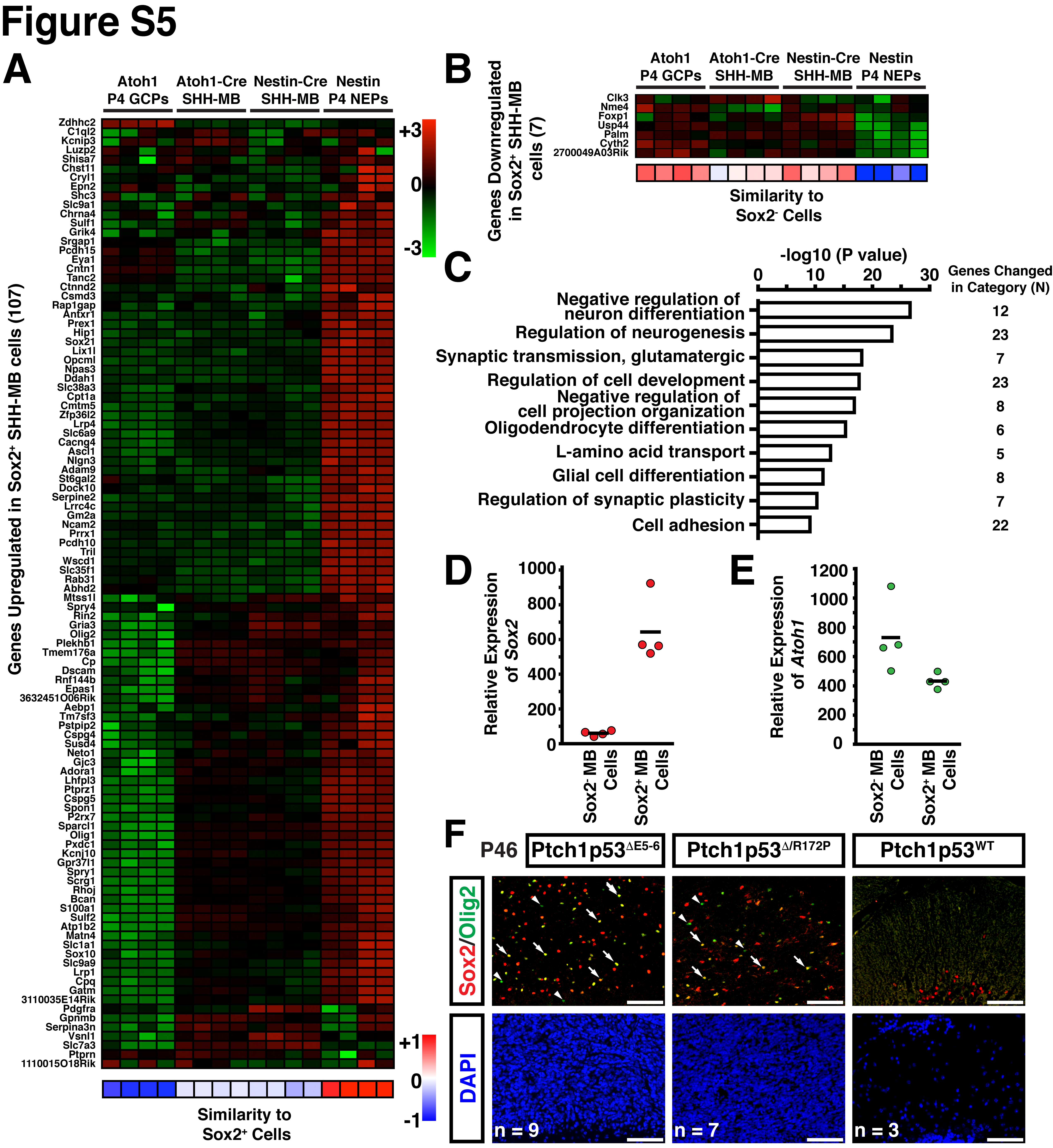

Supplement: vdz027_suppl_Supplementary_Figure_S5 [file vdz027_suppl_supplementary_figure_s5.png]

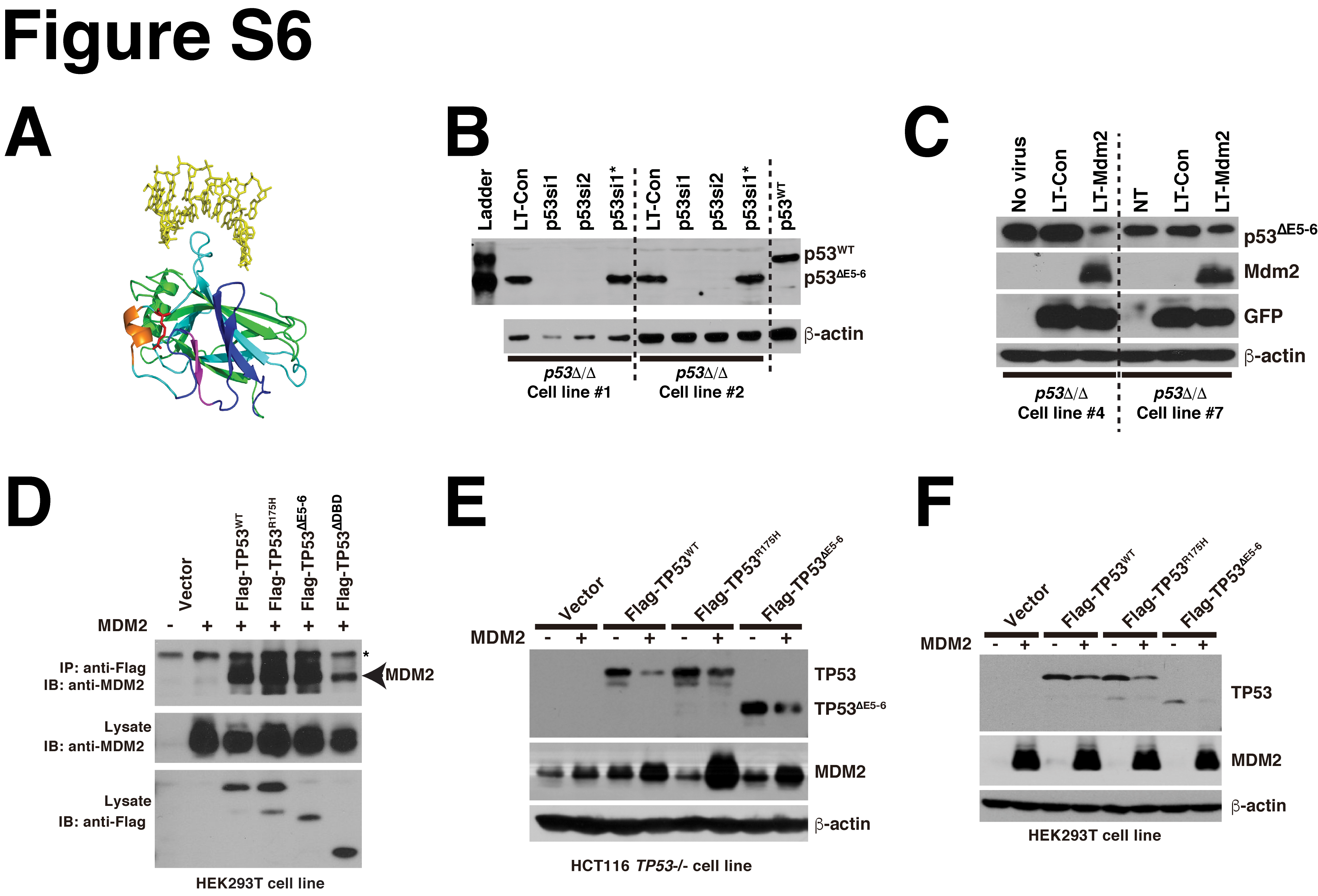

Supplement: vdz027_suppl_Supplementary_Figure_S6 [file vdz027_suppl_supplementary_figure_s6.png]

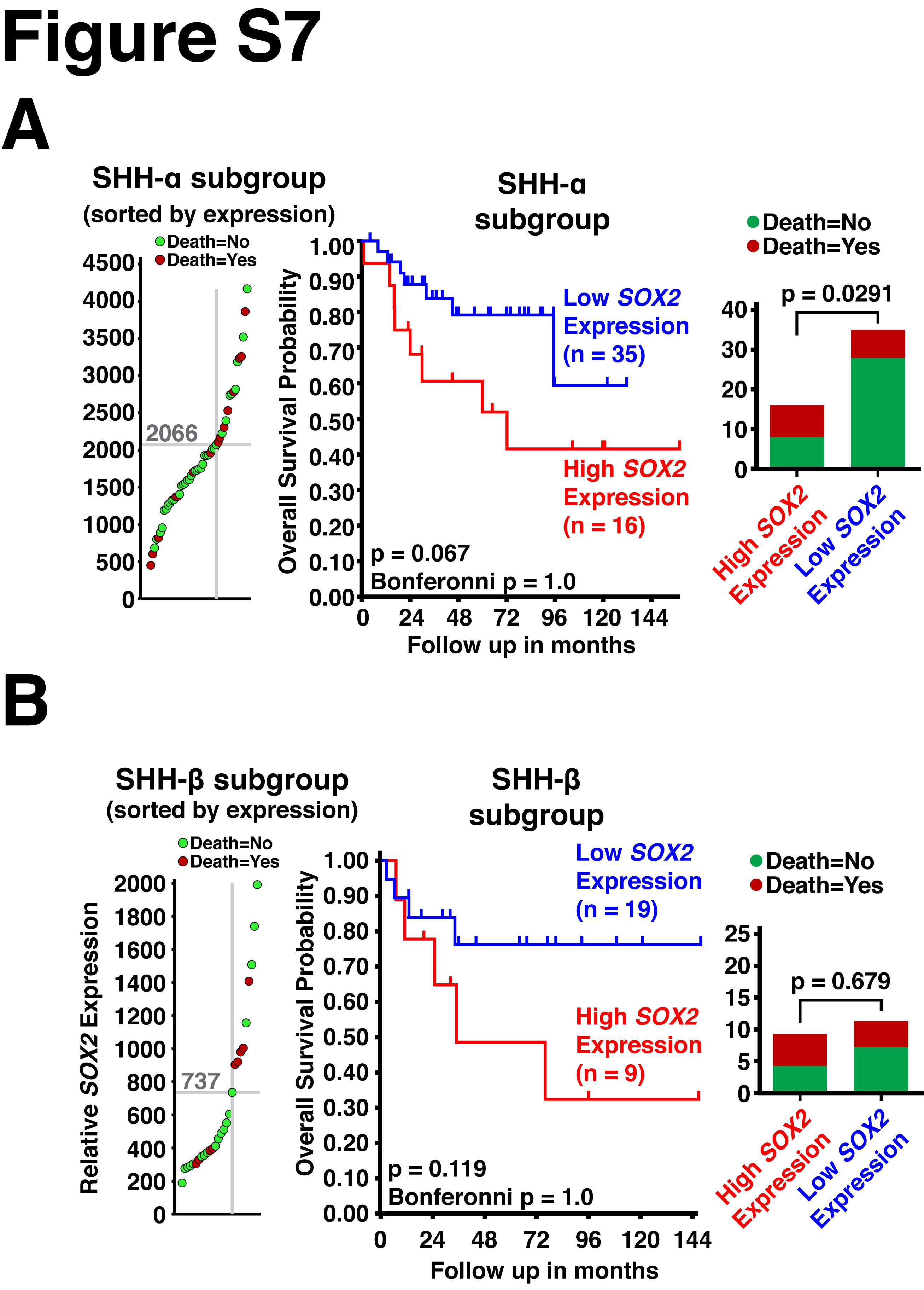

Supplement: vdz027_suppl_Supplementary_Figure_S7 [file vdz027_suppl_supplementary_figure_s7.png]
