# Supplementary material for: Sox2+ cells in Sonic Hedgehog-subtype medulloblastoma resist p53-mediated cell-cycle arrest response and drive therapy-induced recurrence
Source: Neurooncol Adv. 2019 Sep 23;1(1):vdz027. doi: 10.1093/noajnl/vdz027 (PMC6860004; doi:10.1093/noajnl/vdz027)
Supplement: vdz027_suppl_Supplementary_Materials [file vdz027_suppl_supplementary_materials.docx]

**Supplementary Figure captions**

## ­Supplementary Fig. 1. Characterization of SHH-MBs driven by *Ptch1* loss.

(A) The penetrance of MBs from *Ptch1^+/-^p53*^ΔE5-6^, *Ptch1*^+/-^*p53*^R172P^ and *Ptch1*^+/-^*p53*^WT^ mice are quantified. Asymptomatic mice were kept for a minimum of 1-year prior to sacrifice.

(B) Kaplan-Meier survival curves and medulloblastoma free curve for *Ptch1^+/-^p53*^WT/WT^, *Ptch1^+/-^p53*^WT/R172P^ and *Ptch1^+/-^ p53*^WT/∆E5-6^ models are shown (p-value, Log-rank (Mantel-Cox) test).

(C) Representative images of *Ptch1^+/-^p53*^ΔE5-6^, *Ptch1*^+/-^*p53*^R172P^ and *Ptch1*^+/-^*p53*^WT^ MBs show the expression of lineage-specific neuronal and glial markers Synaptophysin, GFAP, Nestin and Olig2. Arrowheads indicate GFAP^+^ tumor cells.

(D-F) Staining of proliferation marker Ki67 and apoptosis marker cleaved Caspase-3 (C-Casp3) (D) are shown. Frequency of proliferation (E) and apoptosis (F) was quantified.

(G) Quantitative RT-PCR of SHH pathway targets was performed in SHH-MBs. Fold change was normalized against adult cerebellum from *Ptch1^+/+^p53*^WT^ littermates (dotted line). *Ptch1*^Exon7-9^ is expressed in mutant and wild-type alleles.

Scale bars: 100 μm.

**­Supplementary Fig.** **2. SHH-MBs retained *p53*^WT^ or *p53*^R172P^, which are reversibly suppressed.**

(A-C) Genomic DNA isolated from matched tails (T) and brain tumors (MB) (A and B) and primary cell lines (C) of *Ptch1^+/-^p53*^ΔE5-6^, *Ptch1*^+/-^*p53*^R172P^ and *Ptch1*^+/-^*p53*^WT^ mice was subjected to PCR analysis for the *p53*^floxE5-6^ (A and C) and *p53*^R172P^ alleles (B and C). Resultant DNA was labeled as: p53^E5-6^ (non-recombined *p53*^floxE5-6^ allele); *p53*^R172P^ (*p53*^R172P^ allele); *p53*^ΔE5-6^ (recombined *p53*^floxE5-6^ allele); and pseudo (*p53* pseudo band). Tails mainly contained un-recombined alleles, whereas MBs predominantly contained recombined alleles. Of note, given the relative positions on the *p53* gene, PCR analysis designed for the non-recombined and recombined *p53*^floxE5-6^ alleles detects the same band for the *p53*^R172P^ allele regardless of recombination (A and C). However, PCR analysis designed for *p53*^R172P^ alleles will not detect the recombined *p53*^floxE5-6^ allele (C).

(D) Targeted Sanger sequencing on the entire coding regions of *p53* gene was performed on control tissues and MBs from *Ptch1^+/-^p53*^R172P^ (n = 6) mice. Aside from G515C, the mutation corresponding to R172P, no other mutation sites were identified on *p53* gene in any of these 6 tumors. Control tissues taken from cerebella of asymptomatic *Ptch1^+/-^p53*^WT/WT^ (CB1) and *Ptch1^+/-^p5*3^E5-6/R172P^ (CB2) mice. One representative sequencing image shows the location of the *p53*^R172P^ mutation, caused by a point mutation, G515C. Targeted Sanger sequencing on the entire coding regions of *p53* gene was performed on additional MBs from *Ptch1^+/-^p53*^WT^ (n = 7) and *Ptch1^+/-^p53*^WT/R172P^ (n = 5) models, no acquired *p53* mutations (other than G515C) was identified (data not shown).

(E) Representative images show the expression of p53, cleaved Caspase-3 (C-Casp3) and Ki67 from *Ptch1^+/-^p53*^∆E5-6^, *Ptch1^+/-^p53*^R172P^ and *Ptch1^+/-^p53*^WT^ MBs 24 hours after a single dose of radiation treatment (4 Gray).

(F) The percentage of Ki67^+^ proliferating cells in non-treated and radiation-treated (4 Gray, 24 Hours) *Ptch1^+/-^p53*^∆E5-6^ and *Ptch1^+/-^p53*^R172P^ MBs was quantified and compared.

(G) Radiation-treated *Ptch1^+/-^p53*^∆E5-6^ and *Ptch1^+/-^p53*^R172P^ MBs were stained with p21, the p53 target responsible for cell cycle arrest. Representative images show that induction of p21 expression is only observed in MBs from *Ptch1^+/-^p53*^R172P^ mice, but not *Ptch1^+/-^p53*^∆E5-6^ mice. Inset indicates the enlarged view of boxed region.

L: Lesion. ML: Molecular Layer. IGL: Internal Granular Layer. Error bars represent Mean ± SEM. Unpaired, two-tailed students t-tests were used to determine p-values. Scale bars: 200 μm.

**­Supplementary Fig**. **3. A 12-day radiation treatment induces potent p53-independent and p53-mediated tumor suppressor activity.**

(A) H&E images from *Ptch1^+/-^p53*^ΔE5-6^, *Ptch1*^+/-^*p53*^R172P^ and *Ptch1*^+/-^*p53*^WT^ P22 cerebella are shown. Boxed regions are shown below. Dashes indicate lesions.

(B and C) Immunofluorescent labeling with proliferative markers Ki67 (B) and BrdU (C) in P22 lesions from *Ptch1^+/-^p53*^ΔE5-6^, *Ptch1*^+/-^*p53*^R172P^ and *Ptch1*^+/-^*p53*^WT^ cerebella are shown.

(D) Comparison of lesion areas in *Ptch1^+/-^p53*^ΔE5-6^, *Ptch1*^+/-^*p53*^R172P^, and *Ptch1*^+/-^*p53*^WT^ cerebella comparing untreated P22 and P46 to treated P35 and P46. Following treatment, an 8-fold reduction in area at P46 was observed in *Ptch1^+/-^p53*^ΔE5-6^ lesions, representing a p53-independent inhibition effect. A 3-fold reduction was observed in *Ptch1^+/-^p53*^R172P^ lesions.

(E) Representative p53 labeling images of *Ptch1^+/-^p53*^ΔE5-6^, *Ptch1*^+/-^*p53*^R172P^, and *Ptch1*^+/-^*p53*^WT^ in P35 lesions following 12-day radiation treatment are shown.

(F and G) Representative H&E images show cellular morphology in P35 *Ptch1^+/-^p53*^ΔE5-6^, *Ptch1*^+/-^*p53*^R172P^, and *Ptch1*^+/-^*p53*^WT^ cerebella after the radiation treatment from P23-P34 (F). Regions in black boxes show lesion cells at higher magnification (below), whereas the region in the red box shows differentiated granule neurons in the IGL for comparison (below, far right). The percentage of radiation-treated lesions containing MB-like undifferentiated cells and IGL-like differentiated neurons was quantified for three *Ptch1*^+/-^ models (G). In heterogeneous clusters, the diagnosis was based on the morphology of the majority of cells within a single cluster.

L: Lesion. ML: Molecular Layer. IGL: Internal Granular Layer. Error bars represent Mean ± SEM. Unpaired, two-tailed students t-tests were used to determine p-values. Scale bars: 200 μm.

**­Supplementary Fig**. **4. Increased proliferation in p53 mutant models leads to tumor recurrence following radiation treatment.**

(A and B) H&E staining of P46 cerebella from *Ptch1^+/-^p53*^ΔE5-6^, *Ptch1*^+/-^*p53*^R172P^ and *Ptch1*^+/-^*p53*^WT^ models (A) are shown. Boxed regions are shown below. The total area per cerebellum (B) was quantified.

(C) Co-labelling of Sox2 and Ki67 was performed on *Ptch1^+/-^p53*^ΔE5-6^, *Ptch1*^+/-^*p53*^R172P^ and *Ptch1*^+/-^*p53*^WT^ lesions at P46.

(D) Quantification of proliferation in P46 lesions demonstrates increased proliferation frequency in *Ptch1^+/-^p53*^ΔE5-6^ and *Ptch1*^+/-^*p53*^R172P^ models following treatment compared to untreated lesions.

(E) H&E images from *Ptch1^+/-^p53*^ΔE5-6^, *Ptch1*^+/-^*p53*^R172P^ and *Ptch1*^+/-^*p53*^WT^ medulloblastoma that formed following 12-day radiation therapy are shown. These tumors are morphologically comparable to untreated SHH-MBs, and feature mitotic figures and nuclear atypia.

(F) Representative images show the expression of Ki67 from *Ptch1^+/-^p53*^∆E5-6^, *Ptch1^+/-^p53*^R172P^ and *Ptch1^+/-^p53*^WT^ MBs that formed following 12-day radiation therapy. Proliferation frequency comparable to untreated tumors is observed.

DAPI was used as a counterstain. Scale bars: 100 μm (A and C), 200 μm (E and F).

**­Supplementary Fig**. **5. Characterization of Sox2 and Olig2 cells in SHH-MBs.**

(A and B) 114 genes associated with Sox2^+^ MB cells (A) and 7 genes associated with Sox2^-^ MB cells (B) were analyzed by supervised clustering within Atoh1^+^ GCPs, SHH-MBs from *Atoh1-cre;Ptch1^flox/flox^* and *Nestin-cre;Ptch1^flox/flox^* mice and Nestin-expressing precursor (NEP) cells. Genome-wide expression profiles of Sox2^+^ MB cells resemble the expression pattern of NEPs, but not Atoh1^+^ GCPs. The relative expression level is displayed by a color scale from high (red) to low (green) as depicted. Relative similarity to the expression in Sox2^+^ (A) or Sox2^-^ (B) cells is shown below using a color scale, from high (red) to low (blue).

(C) Gene Ontology (GO) analysis shows the biological processes associated with the genes that are highly upregulated in Sox2^+^ MB cells, compared to Sox2^-^ MB cells (based on 114 differentially regulated genes). For each pathway, the number of altered genes in this set is shown on the right.

(D and E) *Sox2* (D) and *Atoh1* (E) expression were compared in Sox2^-^ and Sox2^+^ MB cells.

(F) Representative immunofluorescence images co-labelled for Sox2 and Olig2 in P46 lesions in untreated *Ptch1^+/-^p53*^ΔE5-6^, *Ptch1*^+/-^*p53*^R172P^, and *Ptch1*^+/-^*p53*^WT^ cerebella are shown. Arrows and arrowheads indicate Olig2^+^Sox2^+^ and Olig2^+^Sox2^-^ cells, respectively.

DAPI was used as a counterstain. Error bars represent Mean ± SEM. Unpaired, two-tailed students t-tests were used to determine p-values. Scale bars: 100 μm.

## ­Supplementary Fig. 6. Molecular basis of p53^ΔE5-6^ as a marker for stressed and tumor cells.

(A) Structure of the TP53^DBD^-DNA complex. Exons 5 and 6 of *TP53* are colored in cyan and blue, respectively. Two small in-frame deletions observed in human GBMs are colored in orange and purple, respectively; and a hot-spot residue at R175 in exon 5 is colored in red. DNA is shown in yellow.

(B) *p53*^ΔE5-6/ΔE5-6^ malignant glioma cell lines were infected with lenti-viral vectors expressing *p53*-specific siRNAs (*p53*si1 and *p53*si2) or mismatched siRNA (*p53*si1*), or control lenti-virus (LT-Con). Western blot analysis was performed using the cell lysates with the indicated antibodies. Irradiated spleen tissue from *p53*^WT/WT^ animals was used as a positive control for p53 protein.

(C) *p53*^ΔE5-6/ΔE5-6^ mouse glioma cell lines were infected with Mdm2-overexpressing GFP-lenti-virus (LT-Mdm2) or control GFP-virus (LT-Con). Cell lysates were analyzed by Western blot analysis with the indicated antibodies.

(D) Co-immunoprecipitation assay of MDM2 and Flag-tagged TP53-WT or mutant-TP53 proteins (encoded by *TP53*^R175H^*, TP53*^ΔE5-6^*,* and *TP53*^ΔDBD^ mutant alleles). HEK293T cells were co-transfected with the indicated plasmids. Cell lysates were immunoprecipitated with anti-Flag antibody, and probed with anti-MDM2 antibody. The * indicates a non-specific band.

(E and F) Flag-tagged wild type and mutant *TP53* constructs were co-transfected with or without MDM2 plasmid in two human cell lines, HCT116 *TP53*^-/-^ cells (E) or HEK293T cells (F). Cell lysates were subjected to Western blotting analysis with the indicated antibodies.

DBD, DNA-binding domain.

## ­Supplementary Fig. 7. Association of *SOX2*-expression with prognosis in all SHH-MB-α and -β group patients.

(A and B) Scatterplot distribution of *SOX2* gene expression (left), survival analysis (middle) and tumor mortality (right) among all SHH-MB-α (A) and -β (B) group patients. Patients that died due to tumor recurrence/progression are represented by red dots, while other patients are represented by green dots. Multiple analysis were conducted in the *SOX2* expression datasets to find the expression threshold with the greatest significance (lowest p-value) between *SOX2*-high and -low expression values, and the most significant is shown (p-value, Log-rank (Mantel-Cox) test and Bonferroni corrected Log-rank (Mantel-Cox) test), and frequency of tumor mortality at these thresholds was considered (Chi Square test).

**Supplementary Materials and Methods**

**Mouse Model**

Male and female mice carrying a floxed *p53* (*p53*^E5-6^) allele ^1^ were maintained on the mixed C57Bl6/129Svj genetic background. As described previously, the hGFAP-cre mouse ^2^ was initially produced on the FVB background, but was crossed into the mixed background for greater than five generations prior to integration into the *p53* colony ^1^. The *p53*^R172P^ mouse strain ^3^ was crossed into the mixed hGFAP-cre; *p53*^E5-6/E5-6^ background. The *Ptch1^+/-^* model (Goodrich et al., 1997) was maintained on a mixed 129-B6 strain prior to crossing into the hGFAP-cre; *p53*^E5-6/E5-6^ colony. Littermates were used within experimental procedures as often as possible to minimize variance. Control and mutant animals were sacrificed either at experimental endpoints - specifically postnatal day 22 or 46 - or as determined by behavioral and physiological assessment. Asymptomatic animals were monitored for at least 1-year prior to sacrifice. All mice in this study were cared for according to guidelines approved by the Animal Care and Use Committee of the University of Michigan as well as the Institutional Animal Care and Use Committee of Children’s National Medical Center.

**Ionizing radiation Treatment of medulloblastoma (MB) Models**

Matched littermates with MBs were treated with ionizing radiation using a RadSource 2000 with a Mouse Irradiation Fixture and Head Shield (Braintree Scientific Inc.), limiting radiation to the head and neck. Animals diagnosed with neurological symptoms indicative of MBs received either a single dose of 20 Gray (Gy) radiation and were collected 4 hours or 24 hours after treatment. To produce a clinically relevant radiation treatment, matched littermates were treated with 2 Gray per day beginning in their 4^th^ week, between postnatal day 21 and 23, and received radiation 5 times a week for 2 weeks, for a total of 20 Gy, and were collected at either 24 hours or 12 days after the final treatment, or as determined by behavioral and physiological assessment. Treated mice were monitored daily for neurological symptoms.

**Histology, Immunohistochemistry and Immunofluorescence**

Mice were perfused with PBS followed by 4% paraformaldehyde (PFA) for immunohistochemistry, and then paraffin embedded. For molecular assays, mice were perfused with PBS prior to freezing dissected tissues in liquid nitrogen. A subset of control and mutant animals was injected with BrdU, at the concentration of 50 µg per gram body weight, with 3 injections at 2-hour intervals for mice at postnatal day 22 and 46 and were perfused 2 hours after the final injection.

Paraffin embedded tissue was sectioned sagittally at 5 µm thickness, beginning at the midline. Sections were stained at regular intervals (200 μm) with hematoxylin and eosin (H&E) to allow assessment of MBs arising within the mouse models, and all quantification was conducted using at least 3 slides. Stained slides were assessed for the presence of mitotic figures, nuclear molding, and anaplastic cell morphology. Area was measured using ImageJ (1.50i) image processing software (https://imagej.nih.gov/ij/download.html). Tumors were classified using the WHO guidebook for MB as diagnostic criteria, based on the most severe region observed ^4^. Diagnosis was reached based on H&E staining, as well as immunochemical staining with established MB markers. Immunohistochemical stains were conducted on sections adjacent to the H&E stained slides.

Tissues of developing cerebella at P22, P35 and P46 were similarly sectioned and labeled, and lesions were assessed based on previously established criteria: focal or diffuse hyperplasia on the surface of the cerebellum or between folia; hyperchromatic cells with a high nuclei to cytoplasm ratio; atypical or pleomorphic nuclei; and IHC staining revealing proliferative cells or Pax6 positive cells ^5,6^. Similar to end-stage tumors, all H&E quantifications were based on 3 separate slides each separated by 200 μm whenever possible, and IHC labeling was performed on sections adjacent to the largest observed lesions in each specimen.

Immunohistochemistry and immunofluorescence were performed as previously described ^1^. In brief, tissue was rehydrated prior to antigen retrieval using Retrieve-All antigen unmasking system (Covance) then permeabilized in 0.3% Triton X-100 (Sigma-Aldritch). A 5% serum (goat or donkey) blocking buffer was applied for 1 hour, followed by primary antibody overnight at 4°C, and then secondary antibody for 1 hour at room temperature. We used the following primary antibodies: p53 (1:500, rabbit, Leica), Sox2 (1:200, goat, Santa Cruz), NeuN (1:500, mouse, Millipore), Nestin (1:100, mouse, Millipore), cleaved Caspase-3 (1:500, rabbit, Cell Signaling), Ki-67 (1:500, rabbit, Abcam), BrdU (1:500, rat, Abcam), p27 (1:500, Mouse, BD), Olig2 (1:2,000, rabbit, Millipore), GFAP (1:1,000, rabbit, Dako), Synaptophysin (1:200, rabbit, Covance), Pax6 (1:100, Rabbit, Biolegend) and p21 (1:200, mouse, BD). To visualize immunohistochemistry, we used biotinylated secondary antibodies (1:500, Vector Laboratories) with an avidin/biotin-based peroxidase system (Vectastain Elite ABC System, Vector Laboratories) followed by counterstain with hemotoxylin. For immunofluorescent chemistry, we used Alexa488, Alexa555 and Alexa647 conjugated secondary antibodies (1:500, Invitrogen, Life Technologies) followed by counterstain with DAPI. All slides were imaged using an Olympus BX51 or BX53 microscope.

**Primary Cell Culture**

Primary cell cultures were isolated from untreated mutants when mice showed neurological symptoms. Dissected MBs were rinsed in 4ºC PBS prior to transfer into chilled DMEM medium. Tissues were gently minced, and then subjected to enzymatic dissociation using 37ºC Accutase (Innovative Cell Technologies). Following digestion, cells were transferred to serum-free self-renewal medium ^7^. Cells were fed with fresh media ever 3-5 days and passaged every 1-2 weeks, based on observed growth rate, with cells plated onto 6-well ultra-low attachment surface polystyrene plates (Corning). Genomic DNAs were isolated from cells at low passage numbers for genetic characterization.

**TP53-DNA Structure, Plasmid Construction, Cell Culture, Transfection and Coimmunoprecipitation for Mutant P53 Analysis**

Structure of TP53 DNA binding domain (DBD)-DNA complex was based upon the published crystal structure of the TP53DBD-DNA complex ^8^. Each of the *TP53/p53* deletions and mutations used in this study was labeled in the structure of TP53DBD-DNA complex. Plasmids containing full-length human wild-type *TP53* (pC53-SN3) and *MDM2* were gifts from Dr. Yi Sun’s lab at University of Michigan, Ann Arbor. The full length of wild-type *TP53*, *TP53*^R175H^, *TP53*^ΔE5-6^ – the human TP53 version of *p53*^ΔE5-6^*, the* in-frame deletion of Exon 5 and 6) - and *TP53*^ΔE5-8^ – in-frame deletion of Exon 5 to 8 – were amplified by PCR and reconstructed into pCDNA3.1/Flag-His vector (Invitrogen, Carlsbad, CA, USA). The full-length of mouse *Mdm2* was amplified from mouse MEF cells with primers:

MDM2A 5’ – ATGTGCAATACCAAC – 3’

MDM2B 5’ – AGTTGAAGTAAGTTAGCAC – 3’.

Then the *Mdm2* was further re-constructed into pLentilox RSV GFP vector (Core facility of University of Michigan). p53-specific siRNA(s) or mismatched siRNA oligos were constructed into lentiviral vector with H1 promoter (gifts from Dr. Yi Sun’s lab). The targeting sequences chosen for mouse p53 (NM_011640.3) mRNA are:

Sequence 1 5’ – GTACATGTGTAATAGCTCC – 3’

Sequence 2 5’ – CAGTCTACTTCCCGCCATA – 3’

Mismatch 5’ – GTAGATGTCTAATAGCTGC – 3’

Mouse p53 siRNA sequence 1 is from a previously published paper ^9^; sequence 2 is from RANi Codex (<http://cancan.cshl.edu/cgi-bin/Codex/Codex.cgi>). Targeted Sanger sequencing was performed to verify all plasmids. HEK293T and HCT116 *TP53*^-/-^ human cell lines were grown in DMEM containing 10% FBS, 100U/ml penicillin, and 100ug/ml streptomycin at 37°C in 5% CO2. Transfection was performed with SuperFect transfection reagent (Qiagen) and lentivirus was produced by co-transfecting individual plasmids together with pCMVΔR8.9 and pVSV-G plasmids into HEK293T cells. For coimmunoprecipitation assay, HEK293T cells were co-transfected with indicated plasmids. Single transfection with one of the plasmids alone was used as control. 24 hours later, cells were harvested and lysed on ice using 500 ul lysis buffer (50 mM Tris-HCl, pH 7.5, 150 mM NaCl, 1 mM EDTA PH 8.0, 0.5% NP40) in the presence of protease inhibitors. The detergent-soluble fraction was recovered by centrifugation at 4°C for 20 min at 12000 rpm and the supernatants were subjected to immunoprecipitation. 400ul of the supernatant was incubated with mouse anti-Flag antibody (M2, Sigma-Aldrich) and protein G PLUS-Agarose beads (Santa Cruz) with gentle rotation at 4°C for 4 hours. The beads were washed four times with lysis buffer and precipitates were eluted with 2XSDS-PAGE sample buffer and analyzed by Western blotting.

**Western Blot Analysis**

Snap-frozen tissue samples from wild-type control brains and tumors were homogenized in Pierce RIPA Buffer (Thermo Scientific) (10 μl buffer/1 mg tissue), mixed 1:1 with Laemmli Sample Buffer (BioRad) and boiled at 100°C for 8 minutes. Samples were then subjected to SDS-PAGE using the Criterion TGX Precast gels (BioRad) and transferred onto PVDF membranes (Millipore). The membranes were blocked in 5% non-fat milk prepared in TBST and prior to an overnight incubation with primary antibodies at 4°C. Next the membranes were washed with TBST and incubated in horseradish peroxidase (HRP)-conjugated secondary antibodies at room temperature for 1 hour. Signal was detected using Pierce ECL Western Blotting Substrate (Thermo Scientific). The primary antibodies used in this study were as follows: p53 (1:1000, rabbit, Novocastra), M2/Anti-Flag (1:1000, mouse, Sigma), GFP (1:3000, rabbit, Santa Cruz), MDM2 (1:500, mouse, Abcam), β-Actin (1:5,000-10,000, mouse, Sigma-Aldrich). HRP-conjugated secondary antibodies were: anti-mouse (1:5,000-1:10,000, goat, BioRad) and anti-rabbit (1:5,000-1:10,000, goat, BioRad).

**Genotyping**

Genetic analysis of mice in the colony used genomic DNA extracted from tail prior to PCR analysis using the Taq 2X MeanGreen Master Mix (Empyrical BioScience).

*p53*^R172P^

F: 5’ – ACCTGTAGCTCCAGCACTGG – 3’

R: 5’ – ACAAGCCGAGTAACGATCAGG – 3’

*p53*^E5-6^

I6: 5’ – GCTGCAGGTCACCTGTAG – 3’

E7: 5’ – CATGCAGGAGCTATTACACA – 3’

E4: 5’ – TGGGACAGCCAAGTCTGTTA – 3’

*Ptch1*

F: 5’ – TGGGGTGGGATTAGATAAATGCC – 3’

R: 5’ – TGTCTGTGTGTGCTCCTGAATCAC – 3’

hGFAP-Cre

F: 5’ – CCGTTTGCCGGTCGTGGG – 3’

R: 5’ – CGTATATCCTGGCAGCGATC – 3’

**Sanger Sequencing**

Total RNA was isolated from MBs arising within the *Ptch1^+/-^p53*^∆E5-6/∆E5-6^*, Ptch1^+/-^p53*^∆E5-6/R172P^ and *Ptch1^+/-^p53*^WT/WT^ mutants, as well as control cerebellum from both *Ptch1^+/+^p53*^WT/WT^ and *Ptch1^+/-^p53*^WT/WT^ mice, by means of the RNEasy kit (Qiagen), as well as the AllPrep DNA/RNA Mini Kit (Qiagen). cDNA synthesis was performed using QuantiTect Reverse Transcription Kit (Qiagen). The cDNA product, following quality assessment and measurement of concentration, were used to sequence exons of the *p53* allele. The *p53* normal and mutant transcripts, encoding the entire open reading frame, were amplified with primers 1 and 2 and shorter product was prepared combining primers 1 and 4, 3 and 8, and 7 and 2 (Wang et al., 2009). Additionally, *p53* exons 4-8, comprising the p53-DBD (DNA-binding domain), were prepared for sequencing. Amplified PCR products were submitted to the University of Michigan DNA sequencing core or Genewiz L.L.C. for Sanger sequencing. Sequencing primers used with genomic DNA are listed below:

*p53* exon 4

F: 5' - GCTGGTAGGCTGAGAACACA - 3'

R: 5' - ACAGGCTGAAGAGGAACCCC - 3'

*p53* exon 5

F: 5' - CCTTGACACCTGATCGTTAC - 3'

R: 5' - GAGCAAGAATAAGTCAGAAGC - 3'

*p53* exon 6

F: 5' - ACTGGCAGCCTCCCATCTT(C)C - 3'

R: 5' - CTAGAAAGTCAACATCAGTC - 3'

*p53* exon 7

F: 5' - CTAGACTGATGTTGACTTTC - 3'

R: 5' - CTGGGGAAGAAACAGGCTAA - 3'

*p53* exon 8

F: 5' - CTTGTGCTGGTCCTTTTCTTG - 3'

R: 5' - GAGGTGACTTTGGGGTGAAG - 3'

***Ptch1* and *p53* next generation sequencing and variant identification**

Genomic DNAs were isolated from MBs of *Ptch1^+/-^p53*^∆E5-6/∆E5-6^*, Ptch1^+/-^p53*^∆E5-6/R172P^ and *Ptch1^+/-^p53*^WT/WT^ mice, using the AllPrep DNA/RNA Mini Kit (Qiagen). These DNA samples were then analyzed by next generation sequencing to detect point mutants in *Ptch1* and *p53*. First, DNA concentrations were quantified by using a Qubit^R^ dsDNA HS assay kit and a Qubit 2.0 Fluorometer (Life Technologies). The custom primer panel targeting the *Ptch1* and *p53* gene was designed using the AmpliSeq Designer (reference IAD99599_197, Life Technologies). The targeted region (12.38kbp) including the entire *Ptch1*- and *p53*-coding exons and 5 bp exon padding, was amplified by 43 amplicons (length between 125 and 375 bp) distributed in two primer pools, with 100% theoretical coverage. Genomic DNAs were amplified to generate the library using the Ion AmpliSeq Library Kit 2.0 (Life Technologies). The amplified libraries were purified using Agencourt AMPure XP beads (Beckman Coulter, Brea, CA, USA). Before library pooling and sequencing sample preparation, amplified libraries were validated and quantified by qPCR with the Ion Library Quantitation Kit. Emulsion PCR was performed using the Ion OneTouch Instrument (Life Technologies). Enrichment of the template-positive Ion OneTouch 200 ion sphere particles (ISPs, containing clonally amplified DNA) was performed using the Ion OneTouch ES (Life Technologies), according to the manufacturer’s procedures. An ISP quality control was then performed using a Qubit^R^ 2.0 Fluorometer. The template-positive ISPs were loaded on Ion 314 chips and sequenced with an Ion Personal Genome Machine (PGM) System (Life Technologies). Data Ion Torrent reads were collected by the Ion Torrent Suite software v3.6.2, which also sorted the data according to the barcodes. Data collected on the PGM were collated and reanalyzed using the Torrent Suite 3.6.2 using FASTQ files from the Ion Torrent Browser. Sequence alignment was performed with the Ion Torrent Suite on the Ion Torrent Browser (Life Technologies). Single-nucleotide polymorphisms (SNPs) and short insertions and/or deletions calling was performed using the Variant Caller plugin on the Ion Torrent Browser and DNA sequences visualized using the Integrative Genomics Viewer (IGV, version 2.3) from Broad Institute (Cambridge, MA, USA).

**Microarray Analysis**

For gene expression analyses, we used previously published microarray datasets for human SHH MBs (GSE10327, Kool et al., 2008; GSE37418, Robinson et al., 2012; GSE49243, Kool et al., 2014, GSE85218, Cavalli et al., 2017) ^10-13^, mouse Sox2^+^ and Sox2^-^ MB cells (GSE48766, Vanner et al., 2014) ^14^, and mouse Atoh1^+^ and NEP cells plus mouse MB tumors (GSE50824, Li et al., 2013) ^15^. For data analysis and data visualization we have used the R2: Genomics Analysis and Visualization Platform (<http://hgserver1.amc.nl/cgi-bin/r2/main.cgi>). The Kaplan scanner tool in R2 was used to find the best cut-off in expression within a certain group of tumors for which the Kaplan Meier analysis gives the lowest log rank p-value. We performed the Bonferroni correction and reported a Bonferroni adjusted p-value for multiple testing as multiple tests were performed to find the lowest p-value. The plots that we showed in Figure 6 were the plots with lowest p-values and other cut-offs gave higher p-values. Therefore, the expression threshold between each subgroup varied. However, as overall expression levels are also different between subgroups, it still makes sense to use different cut-offs. R2 is a microarray analysis and visualization platform that is freely available online: <http://r2.amc.nl>.

**Quantitative RT-PCR for SHH and p53 pathway target expression in Tumors**

Extracted RNA (see above) was used to produce cDNA for qRT-PCR (QuantiTect Reverse Transcription Kit, Qiagen). Template cDNA was combined with QuantiTect SYBR Green Master Mix (Qiagen) and the following primers for qRT-PCR:

*p21*

F: 5’ - CACAGCTCAGTGGACTGGAA - 3’

R: 5’ - ACCCTAGACCCACAATGCAG - 3’

*Puma*

F: 5’ - GCGGCGGAGACAAGAAGA - 3’

R: 5’ - AGTCCCATGAAGAGATTGTACATGAC - 3’

*Gli1*

F: 5’ - GCTCCGCAAACACGTGAAGA - 3’

R: 5’ - GCCGGATCCTCCTTCCCTTT - 3’

*Gli2*

F: 5’ - TCTAGTCCACGTGTGACCCC - 3’

R: 5’ - AGGTCCGAATCATGCGTTGT - 3’

*N-Myc*

F: 5’ - AGGAAGCACTCCCCCATATT - 3’

R: 5’ - CCGCCGAAGTAGAAGTCATC - 3’

*Olig2*

F: 5’ - ATGCACGACCTCAACATCGCCA - 3’

R: 5’ - ACCAGTCGCTTCATCTCCTCCA - 3’

*Twist1*

F: 5’ - GATTCAGACCCTCAAACTGGCG - 3’

R: 5’ - AGACGGAGAAGGCGTAGCTGAG - 3’

*Twist2*

F: 5’ - CAGCAAGATCCAGACGCTCAAG - 3’

R: 5’ - ACACGGAGAAGGCGTAGCTGAG - 3’

*Wip1*

F: 5’ - TCACAGTGGACCTGTCAGAAGG - 3’

R: 5’ - AGAGTGTGGACACTGGTGTCTG - 3’

*Trim24*

F: 5’ - CTAAATGGGCTGTTTCCAGTGGC - 3’

R: 5’ - GGTGTTGGTCACAGGAGAAGCA - 3’

*Mdm4*

F: 5’ - GCTCTCGCACAGGATCACACTA - 3’

R: 5’ - ATGTCGTGAGGTAGGCAGTGTG - 3’

*GapDH*

F: 5’ - ACCCAGAAGACTGTGGATGG - 3’

R: 5’ - CACATTGGGGGTAGGAACAC - 3’

All PCR experiments were conducted using Applied Biosystems 7500 Real-Time PCR System. Results were analyzed by comparing ∆C_T_ between targets, then normalizing to the expression of the housekeeping gene GADPH. All samples were run in triplicate to minimize internal variance.

**Statistical Analysis**

Kaplan-Meir survival curves were prepared using Prism, and included mice sacrificed due to brain tumor, body tumor and veterinary concerns, as well as aged control mice, and compared using the Mantel-Cox test. Histology and whole mount area quantification was determined using ImageJ (NIH). Cell number was counted manually and verified with ImageJ cell counter. Whenever possible, quantification was conducted using multiple replicates taken from discrete regions of the brain as well as multiple sections. Student’s 2-tailed t-test was used for the comparison of areas, cell numbers and frequency. Where multiple groups were present, ANOVA was used with Bonferroni’s multiple comparisons test to calculate p-value. Data were presented as Mean + SEM (standard error of the mean), and p < 0.05 was considered to be statistically significant.

**­Supplementary References**

**1.** Wang Y, Yang J, Zheng H, et al. Expression of mutant p53 proteins implicates a lineage relationship between neural stem cells and malignant astrocytic glioma in a murine model. *Cancer Cell.* 2009; 15(6):514-526.

**2.** Zhuo L, Theis M, Alvarez-Maya I, Brenner M, Willecke K, Messing A. hGFAP-cre transgenic mice for manipulation of glial and neuronal function in vivo. *Genesis.* 2001; 31(2):85-94.

**3.** Liu G, Parant JM, Lang G, et al. Chromosome stability, in the absence of apoptosis, is critical for suppression of tumorigenesis in Trp53 mutant mice. *Nat Genet.* 2004; 36(1):63-68.

**4.** Louis DN, Ohgaki H, Wiestler OD, Cavenee WK. *WHO Classification of Tumors of the Central Nervous System*. Lyon IARC; 2007.

**5.** Mille F, Tamayo-Orrego L, Levesque M, et al. The Shh receptor Boc promotes progression of early medulloblastoma to advanced tumors. *Developmental cell.* 2014; 31(1):34-47.

**6.** Malek R, Matta J, Taylor N, Perry ME, Mendrysa SM. The p53 inhibitor MDM2 facilitates Sonic Hedgehog-mediated tumorigenesis and influences cerebellar foliation. *PLoS One.* 2011; 6(3):e17884.

**7.** Molofsky AV, Pardal R, Iwashita T, Park IK, Clarke MF, Morrison SJ. Bmi-1 dependence distinguishes neural stem cell self-renewal from progenitor proliferation. *Nature.* 2003; 425(6961):962-967.

**8.** Cho Y, Gorina S, Jeffrey PD, Pavletich NP. Crystal structure of a p53 tumor suppressor-DNA complex: understanding tumorigenic mutations. *Science.* 1994; 265(5170):346-355.

**9.** Dirac AM, Bernards R. Reversal of senescence in mouse fibroblasts through lentiviral suppression of p53. *J Biol Chem.* 2003; 278(14):11731-11734.

**10.** Kool M, Jones DT, Jager N, et al. Genome sequencing of SHH medulloblastoma predicts genotype-related response to smoothened inhibition. *Cancer Cell.* 2014; 25(3):393-405.

**11.** Kool M, Koster J, Bunt J, et al. Integrated genomics identifies five medulloblastoma subtypes with distinct genetic profiles, pathway signatures and clinicopathological features. *PLoS One.* 2008; 3(8):e3088.

**12.** Robinson G, Parker M, Kranenburg TA, et al. Novel mutations target distinct subgroups of medulloblastoma. *Nature.* 2012; 488(7409):43-48.

**13.** Cavalli FMG, Remke M, Rampasek L, et al. Intertumoral Heterogeneity within Medulloblastoma Subgroups. *Cancer Cell.* 2017; 31(6):737-754 e736.

**14.** Vanner RJ, Remke M, Gallo M, et al. Quiescent sox2(+) cells drive hierarchical growth and relapse in sonic hedgehog subgroup medulloblastoma. *Cancer Cell.* 2014; 26(1):33-47.

**15.** Li P, Du F, Yuelling LW, et al. A population of Nestin-expressing progenitors in the cerebellum exhibits increased tumorigenicity. *Nat Neurosci.* 2013; 16(12):1737-1744.
